# c-Myb Binding Sites in Haematopoietic Chromatin Landscapes

**DOI:** 10.1371/journal.pone.0133280

**Published:** 2015-07-24

**Authors:** Mads Bengtsen, Kjetil Klepper, Sveinung Gundersen, Ignacio Cuervo, Finn Drabløs, Eivind Hovig, Geir Kjetil Sandve, Odd Stokke Gabrielsen, Ragnhild Eskeland

**Affiliations:** 1 Department of Biosciences, University of Oslo, Oslo, Norway; 2 Department of Cancer Research and Molecular Medicine, Norwegian University of Science and Technology, Trondheim, Norway; 3 Department of Informatics, University of Oslo, Oslo, Norway; 4 Department of Tumour Biology, The Norwegian Radium Hospital, Oslo University Hospital, Oslo, Norway; 5 Department of Medical Informatics, The Norwegian Radium Hospital, Oslo University Hospital, Oslo, Norway; University of Connecticut, UNITED STATES

## Abstract

Strict control of tissue-specific gene expression plays a pivotal role during lineage commitment. The transcription factor c-Myb has an essential role in adult haematopoiesis and functions as an oncogene when rearranged in human cancers. Here we have exploited digital genomic footprinting analysis to obtain a global picture of c-Myb occupancy in the genome of six different haematopoietic cell-types. We have biologically validated several c-Myb footprints using c-Myb knockdown data, reporter assays and DamID analysis. We show that our predicted conserved c-Myb footprints are highly dependent on the haematopoietic cell type, but that there is a group of gene targets common to all cell-types analysed. Furthermore, we find that c-Myb footprints co-localise with active histone mark H3K4me3 and are significantly enriched at exons. We analysed co-localisation of c-Myb footprints with 104 chromatin regulatory factors in K562 cells, and identified nine proteins that are enriched together with c-Myb footprints on genes positively regulated by c-Myb and one protein enriched on negatively regulated genes. Our data suggest that c-Myb is a transcription factor with multifaceted target regulation depending on cell type.

## Introduction

c-Myb is a key regulatory transcription factor (TF) essential for normal adult haematopoiesis [1–4]. It is a TF highly expressed in haematopoietic stem cells and progenitors, and plays a direct role in lineage commitment where its downregulation is associated with haematopoietic maturation and differentiation of both myeloid and B and T lymphoid progenitor cells [5–8]. Clinical studies have revealed strong links between c-Myb aberrations and human cancer. The *MYB* gene is frequently rearranged in several human neoplasias, such as acute myelogenous leukaemia, melanoma, and breast, colon and pancreatic carcinoma [9–11]. In some cancers this involves amplification of the *MYB* gene and increased c-Myb expression. The expression level of c-Myb is also tightly controlled by specific miRNAs [12,13]. A recent report identified a group of tumour suppressor miRNAs with reduced abundance in leukaemia cells from patients with T-cell acute lymphoblastic leukaemia (T-ALL) [14]. Since these miRNAs all converged on *MYB*, their downregulation caused increased c-Myb expression in the T-ALL patients. On the other hand, studies of a knockdown allele of *Myb* in mice have shown that reduced levels of c-Myb can also severely perturb haematopoiesis [6–8,15]. The emerging picture from these studies is that the level of c-Myb is critical for proper function in haematopoietic tissue, and that only a two-fold up- or down-regulation may have dramatic biological effects. In order to understand the biological effects of altered c-Myb levels, it is important to know the c-Myb binding sites and target genes in haematopoiesis and cancer.

Although some studies have identified potential target genes by knockdown or induced expression of c-Myb [1,5,16–25], very few genome-wide studies of c-Myb enrichment are available. Chromatin immunoprecipitation followed by high-throughput DNA sequencing (ChIP-seq) relies on good antibodies and this is where c-Myb may have had limitations. A ChIP-seq dataset mapping c-Myb binding sites of an ER-MYB fusion protein in myeloid progenitor cells has been reported [5]. However, a severely truncated c-Myb part was immunoprecipitated lacking important functional regions, and we cannot exclude that c-Myb binding could be sterically influenced by the large ER part of the fusion [5]. ENCODE has published one c-Myb ChIP-seq dataset from murine MEL cells from the Snyder laboratory. However no published study of this dataset is available [9,14]. A recent paper reported c-Myb ChIP-seq datasets from MOLT-3 and Jurkat cells, but the authors limited their analysis to studying an oncogenic super-enhancer [26].

Antibody independent methods offer an alternative way of mapping binding of proteins to chromatin, such as DamID or chromatin accessibility analysis that maps DNA occluding factors with nucleases. DNase I footprinting has been used as a method to study DNA protection for over 35 years [27]. With recent developments in sequencing technology, mapping of nuclease-protected DNA can be used genome-widely at single base pair resolution. Digital genomic footprinting (DGF) uses massively parallel sequencing of DNase I treated cells to map proteins associated with specific DNA sequences on a global scale [28–32]. The identity of the factors bound is deduced from comparing the DNA sequence within the footprint with known sequence recognition patterns of different TFs.

In this work, we have exploited this alternative DGF strategy to obtain a global picture of c-Myb occupancy in the human genome. We have investigated c-Myb binding in six different haematopoietic cell-types using DGF and biologically validated the c-Myb footprints using c-Myb knockdown data, reporter assays and DamID analysis. We show that the predicted c-Myb specific binding sites vary strongly among haematopoietic cell-types, but that there is a set of c-Myb footprints that are common to all cell-types analysed. We identify c-Myb footprints for both up- and down-regulated targets in K562 cells c-Myb is a TF of critical importance for correct haematopoietic development and our predictions show that c-Myb has differential occupancy depending on cell type reflecting its role in both lineage commitment and differentiation.

## Results

### Genome-wide prediction of c-Myb footprints

**Fig 1:**
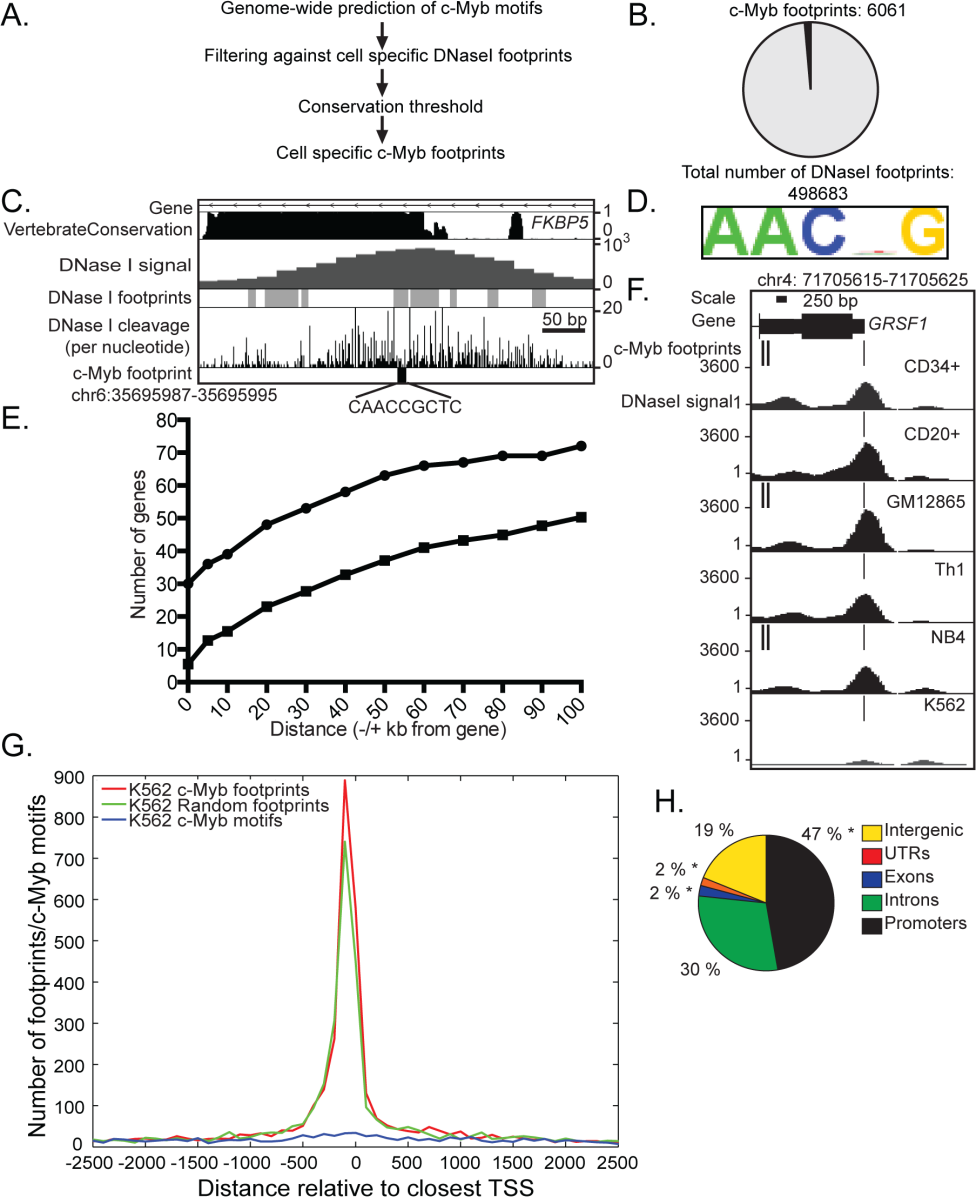
Identification of c-Myb footprints. (A) Workflow for identification of c-Myb footprints. (B) A pie chart representing the number of c-Myb footprints identified compared to the total number of footprints in K562 cells. (C) An illustration from Motiflab showing a c-Myb motif located at the start of the first intron of the gene *FKBP5* that overlaps with a DNase I footprint with a high conservation value in K562 cells, illustration modified. The coordinates for the c-Myb footprint is shown above the illustration, and to the right are the signal intensity for the DNase I datasets, in addition to a conservation score. (D) The binding motif enriched in c-Myb footprints in K562 cells. (E) Graph of the presence of c-Myb footprints and the distances to the 100 most regulated genes upon KD in K562 cells (dots) or a selection of 100 random genes (squares), an average of ten repetitions. Zero base pair indicates that the c-Myb footprints are found inside the gene body (F) A c-Myb footprint at the TSS of GRSF1 gene mapped in all six cell-types analysed. Coordinates for c-Myb footprint are shown above, and to the left are the signal intensity for DNase I datasets. (G) Position of c-Myb footprints, and random selections of DNase I footprints and c-Myb motifs, respectively, around ENSEMBL annotated TSS in K562 cells. (H) Distribution of c-Myb footprints at annotated genes, promoters and intergenic regions in K562 cells. *Overlapping significantly higher with c-Myb footprints than expected by random sampling of K562 DNase I footprints (p' < 5x10^-2^).

DGF is a powerful method to identify nucleotides protected by proteins at a genome-wide scale independent of antibodies [29–32]. To map changes in c-Myb occupancy during haematopoiesis, we used DGF to generate maps of c-Myb footprints with nucleotide resolution (Fig 1A). We selected haematopoietic cell-types where c-Myb is expressed at different levels: c-Myb is highly expressed in haematopoietic stem cells [33] and expressed at lower level in CD4+ T-helper cells [34] and B cells [35,36]. c-Myb is also highly expressed in most cases of leukaemia [10]. We collected available DNase I footprint datasets in six different human cell-types from three healthy donors (CD34+ (mobilized), CD20+ and Th1 cells), transformed B-lymphocytes (GM12865) and two cancer cell-types where c-Myb is upregulated: erythroleukaemia (K562) and promyelocytic leukaemia (NB4) [31].

To predict potential c-Myb binding sites (c-Myb footprints), we first scanned the human genome with MotifLab [37] using four c-Myb motifs from the TRANSFAC database [38]. We identified more than 19 million c-Myb motif instances and filtered these against cell-specific DNase I footprints from the six different cell-types (Fig 1A) [31,39]. We decided that a c-Myb motif was regarded as occupied in each respective cell type if 90% of the motif overlapped a DNase I footprint. We found that between 0.14–0.3% of the total c-Myb motifs overlapped DNase I footprint signals in the six cell-types analysed (S1 Table).

It has previously been reported that factor specific DNase I footprints show a higher evolutionary conservation than immediately adjacent sequences and that these correspond with ChIP-seq signals [30,32,40]. We utilized information on weighted average conservation score (phastCons46wayPlacental) [41] to weigh each position in the footprint according to the information content of the corresponding column in the c-Myb motif. Sites that scored below 0.22 were discarded from further consideration. In total, we identified between 6061 and 12338 evolutionary conserved c-Myb footprints depending on the cell type (Fig 1B, S1A–S1F Fig and S1 Table). This is illustrated in Fig 1C where a c-Myb footprint in K562 cells fell within the first intron of c-Myb regulated *FKBP5* gene [1]and falls within an evolutionary conserved region. In all six cell-types, the weighted average conservation for each predicted motif instance are elevated for all genome-wide c-Myb footprints compared to all identified c-Myb motifs (S1A–S1F Fig).

We scanned the remaining 6061 c-Myb footprints in K562 cells with ChIPMunk [42] and identified a five nucleotide signature resembling the core c-Myb binding motif (Fig 1D). A similar c-Myb binding motif was identified in the other five cell-types (S1G–S1K Fig). This close resemblance of the five nucleotide signatures was expected as our analysis started with four c-Myb motifs from TRANSFAC database [38].

In order to evaluate the relevance of this collection of deduced c-Myb binding sites, we examined the correlation of the identified c-Myb footprints with a list of c-Myb target genes derived from c-Myb knockdown in K562 cells [1]. Seven of the ten most down-regulated genes (*KCNH2*, *LMO2*, *MYB*, *MYADM*, *STNM3*, *EPCAM* and *GRSF1*) had c-Myb footprints localized within the gene locus. For the gene GLUL, a c-Myb footprint was located 19 kilo bases (kb) downstream of the gene (S2 Table). Two target genes had no conserved c-Myb footprint present. Mapping c-Myb-footprints at the majority of these genes is consistent with c-Myb being involved in the activation of these. For genes being repressed by c-Myb, we identified c-Myb footprints in five of the ten most upregulated genes: within the gene locus for *GDF15*, *MKRN1*, *MRAP2*, *LEPR*, *CPEB4*. For two upregulated genes *SH3BGRL3* and *SLC30A10*, c-Myb footprints were identified 4 kb and 15 kb upstream respectively (S3 Table). The presence of conserved c-Myb footprints at a high fraction of gene loci that are most affected by c-Myb silencing suggests a role of c-Myb in direct regulation of these genes in K562 cells. We further extended this analysis to the 100 most up- or down-regulated genes upon c-Myb knockdown in K562 cells (Fig 1E) [1]. We find that 30% of these genes had conserved c-Myb footprints within the gene body. A total of 39% of the top 100 c-Myb target genes had a c-Myb footprint located +/- 10 kb from the gene body. Most cis-acting regulatory elements are found within 10–200 kb of their target genes [43]. By extending our analysis to +/-100 kb, we detected c-Myb footprints at 72% of the top 100 genes. The remaining 28% of genes had no c-Myb footprints and may not be direct targets of c-Myb, or these genes may be regulated by c-Myb at binding sites that are not conserved. Additional alternatives may be that c-Myb binds to a DNA sequence motif different to the four TRANSFAC motifs used in this analysis, or indirect association of c-Myb with chromatin through interaction with another bond TF or co-factor. We also generated a graph displaying average of random sample of 100 genes repeated ten times which show a marked decrease in genes with c-Myb footprints (Fig 1E). For example, only 5.5% of these random genes had conserved c-Myb footprints within the gene locus, and 15.5% random genes had a c-Myb footprint located +/- 10 kb from the gene body.

We found that c-Myb footprints show a high degree of cell specificity, but there is also a common core of c-Myb footprints that could be detected in all six cell-types, suggesting that c-Myb may control both common functions and specific gene programs. One example is a c-Myb footprint that maps to the transcription start site (TSS) of the *GRSF1* gene in all six cell-types (Fig 1F). Nonetheless, two other c-Myb footprints in the first intron of *GRSF1* are only present in three cell-types (CD34+, GM12865 and NB4), suggesting a complex combination of general and cell type dependent control by c-Myb.

We analysed the global distribution of c-Myb footprints and found that between 10 and 15% (900–1300 footprints depending on cell type) map to the promoter directly upstream of TSS (Fig 1G and S2A–S2E Fig). In comparison, a random sample of the same number of predicted c-Myb motif hits in the respective cell-types showed far less preference for mapping close to the TSS. When we carried out the same analysis with the same number of randomly selected DNase I footprints, we found a similar TSS localization as with the c-Myb footprints, but with a slightly lower frequency directly upstream of TSS. Our analysis show that c-Myb footprints and randomly selected DNase I footprints follow a common pattern at TSS.

On a global level, we found that c-Myb footprints in K562 cells were located more in promoter regions (47%) and introns (30%) compared to intergenic regions (19%) (Fig 1H). When we compared this across the other cell-types, a large proportion of c-Myb footprints was present at promoters, with Th1 and CD20+ cells having over 60% of the c-Myb footprints located in these regions. In comparison, the percentages of c-Myb footprints at promoters were less (43–48%) in CD34+, GM12865, NB4, and K562 cells with more footprints in introns (28–31%) and intergenic sequences (18–21%) (Fig 1H and S2F Fig). However, when we compared our analysis with random sampling of DNase I footprints in K562 cells, c-Myb footprints overlapped significantly more with exons (with a normalised ratio, r, of 3.47), UTR regions (r = 1.27), and promoters (r = 1.10) than would be expected by random sampling of DNase I footprints (FDR-corrected p-value, p' < 0.05) (S4 Table). In the five other cell-types, c-Myb footprints were located significantly more in exon regions (with normalised ratios ranging from 2.98 to 3.96, p' < 0.05) (S4 Table) and 3'-UTR regions than expected by random sampling of DNase I footprints (with normalised ratios ranging from 1.10 to 1.44, p' < 0.05). For NB4 and GM12865 cells there was a slightly higher localization in promoter regions (normalised ratios 1.04 and 1.08, respectively, with p' < 0.05) (S2F Fig and S4 Table). Therefore, we conclude that c-Myb footprints differ from random sampling of DNase I footprints by locating more in exons than at promoters although the total number of c-Myb footprints in promoters is higher in all six cell-types.

### Validation of predicted c-Myb footprints

**Fig 2:**
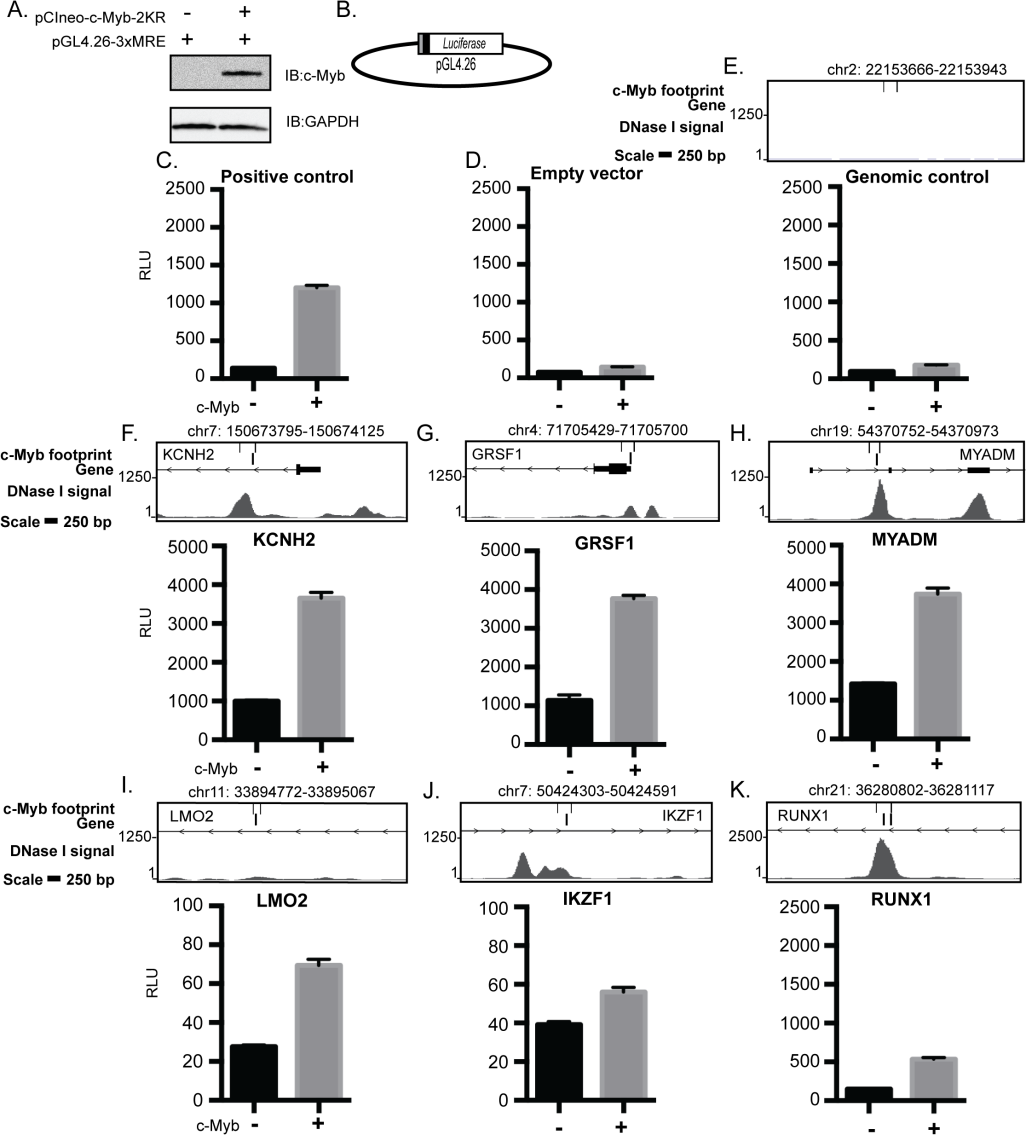
c-Myb enhances transcription from genomic elements with c-Myb footprints. Luciferase-based reporter assay to study the responsiveness of genomic regions, with one or more c-MYB footprints mapped. (A) Representative Immunoblot of CV1 cells transfected with reporter plasmid and-/+ c-Myb. (B) Map of the pGL4.26 vector used for the luciferase assays. The grey box illustrates the genomic fragment containing a c-Myb footprint or control region. The black box illustrates the minimal promoter. (C) Positive control with three MREs. (D-E) Negative controls, (F-K) genomic loci identified to contain c-Myb footprints. The upper panels show the genomic region in the UCSC browser (hg19) presenting DNase I signals, c-Myb footprints and oligos for selected region. The coordinates for the c-Myb footprint are shown above the illustration. The values are the average from three independent experiments-/+ SEM.

To test whether a selection of c-Myb footprints is bound by c-Myb and in turn causes activation of the neighbouring gene, we performed transient reporter assays in CV-1 cells. We used a sumoylation-deficient c-Myb mutant (c-Myb-2KR) to ensure active c-Myb (Fig 2A) [1,44,45]. We selected nine regions containing c-Myb footprints that mapped to genes being activated by c-Myb (*KCNH2*, *LMO2*, *MYADM*, *GRSF1*, *IKZF1*, *SENP1*, *DUS3L*, *RABEPK* and *DCAF7*) (S2 and S5 Tables) in K562 cells [1]. Furthermore, we included four other K562 c-Myb footprints located in proximity of or within the gene loci not known to be regulated by c-Myb in K562 cells *(RUNX1*, *RUNX2*, *KB-1458E12*.*1 and C10orf55)*. Each amplified sequence (average 280 base pairs (bp)) spanning a c-Myb footprint was inserted into a luciferase reporter plasmid upstream of the minimal SV40-promoter (Fig 2B). As negative control we selected a genomic region on chromosome 2 that lacked c-Myb footprints. This control reporter showed only a marginal response similar to the empty vector (Fig 2D and 2E). Several of the selected regions (*KCNH2*, *MYADM*, *GRSF1*, *SENP1*, *RABEPK*, *DCAF7 and C10orf55)* showed a c-Myb response equal to or higher than the 3xMRE positive control (Fig 2F, 2G and 2H, S3A, S3B, S3D, S3E and S3G Fig). The base level differed largely between the constructs as expected since they span a larger segment than the just c-Myb footprint. A weaker response was measured for c-Myb footprints at the loci of *LMO2*, *IKFZ1*, *RUNX1*, *DUS3L*, *KB-1458E12*.*1* and *RUNX2* (Fig 2I, 2J, 2K and S3C, S3F and S3H Fig). These data confirmed that most of the selected c-Myb footprints, taken out of their normal context, confer c-Myb response consistent with c-Myb being capable of binding to the footprints and able to enhance transcription of the neighbouring gene (Fig 2 and S3 Fig).

**Fig 3:**
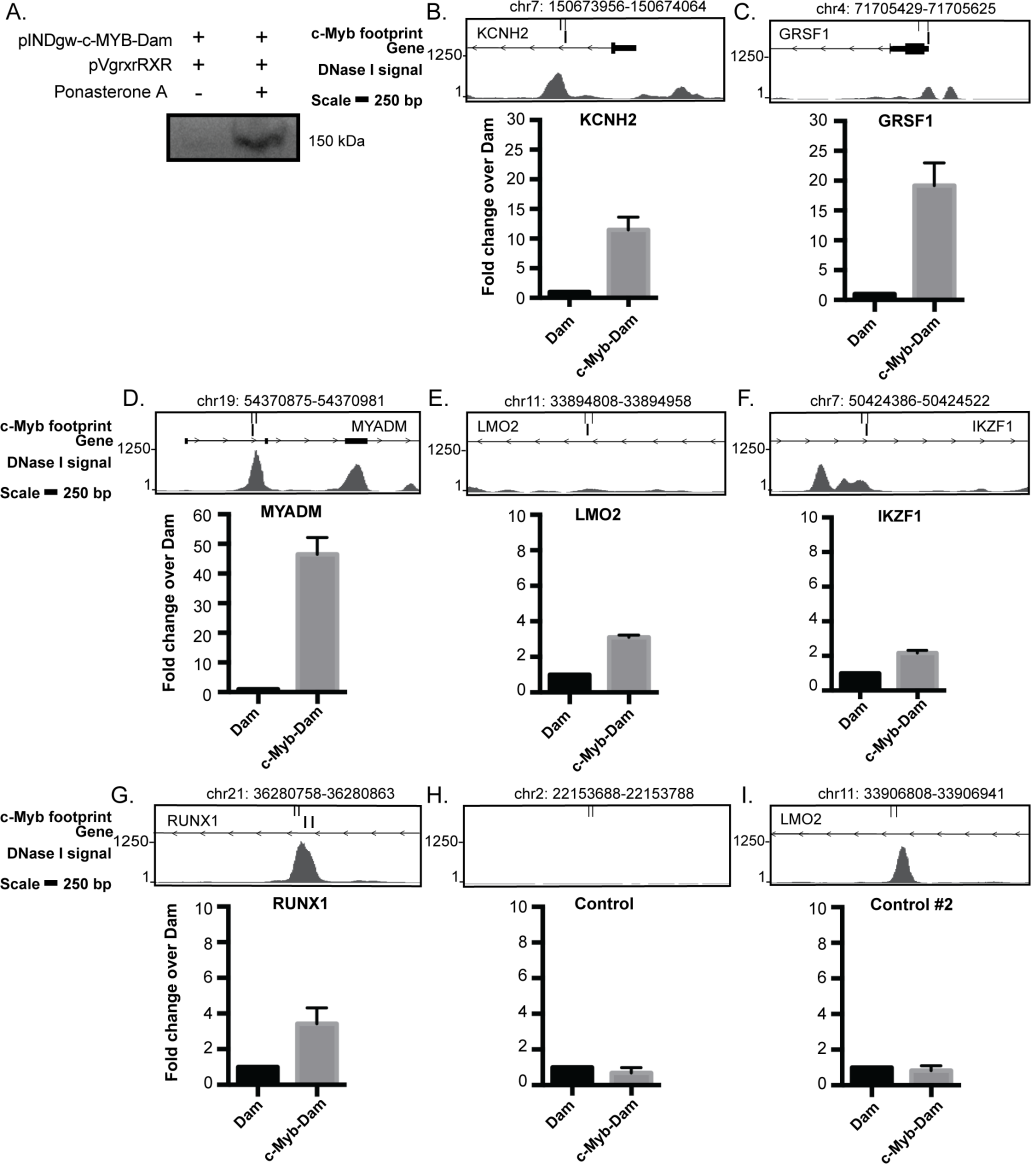
Validation of c-Myb footprints by DAMID. (A) Expression of the Flag-c-Myb-Dam construct. K562 cells were transfected with a plasmid encoding the c-Myb-Dam together with the activator plasmid pVgRXR and treated with 2 uM of Ponasterone A. Expression of the fusion construct was detected by immunoblotting against Flag-tag. (B-J) Association of the control Dam and c-Myb-Dam at specific loci containing one or more c-Myb footprints quantified with qPCR and normalised to Dam. The upper panels show the genomic region in the UCSC browser (hg19) presenting DNase I signals, c-Myb footprints and oligos for qPCR. The coordinates for the c-Myb footprint are shown above the illustration. The values represent the average from two independent cell lines-/+ SEM.

In order to further validate the deduced c-Myb footprints, we performed a DamID analysis in K562 cells (S4A Fig) [46,47]. DNA adenine methyltransferase (Dam) was fused to full-length c-Myb, and we generated a pool of stably transfected cells that express trace amounts of Dam or c-Myb-Dam. It is critical to keep the Dam and Myb-Dam expression low to avoid too high background methylation. This precludes direct detection of the trace levels by normal Western blotting. We used an ecdysone-inducible promoter to detect the c-Myb-Dam expression and performed transient transfection together with the pVgRXR vector encoding the ecdysone receptor in K562 cells and induced expression by the ecdysone analog Ponasterone A [48]. A clear induction of the fusion protein was observed (Fig 3A). To rule out the effects of random integration of transgenes, we used two stable K562 pool cell lines for Dam and Dam-Myb derived at different time points. Finally, we used qPCR with oligos spanning selected c-Myb footprints to map c-Myb binding at these sites and compared the signals to those obtained with the Dam only cells.

To monitor c-Myb binding at c-Myb footprints in K562 cells, we monitored DamID signals by q-RT-PCR at six selected regions measured in the reporter assay (Fig 2), and in addition two controls and three other regions where we had detected c-Myb footprints. At two selected control loci without predicted c-Myb footprints we detected less c-Myb-Dam binding relative to Dam alone (Fig 2H and 2I). We detected c-Myb binding at five gene loci with c-Myb footprints that also showed response in the reporter assay (*KCNH2*, *LMO2*, *MYADM*, *GRSF1* and *RUNX1)* (Figs 2 and 3). Interestingly, we observed weak enrichment of the c-Myb footprint at the *IKZF1* locus, which showed only marginal response in the reporter assay (Figs 2J and 3F). We also detected binding of c-Myb-Dam over Dam alone at three other loci (*CBFA2T3*, *BHLHE40* and *PA2G4)* (S4B–S4D Fig). These results show that almost all loci with predicted c-Myb footprints that were tested by DamID are bound by c-Myb-Dam in K562 cells.

### Histone modifications and transcription factors associated with c-Myb footprints

**Fig 4:**
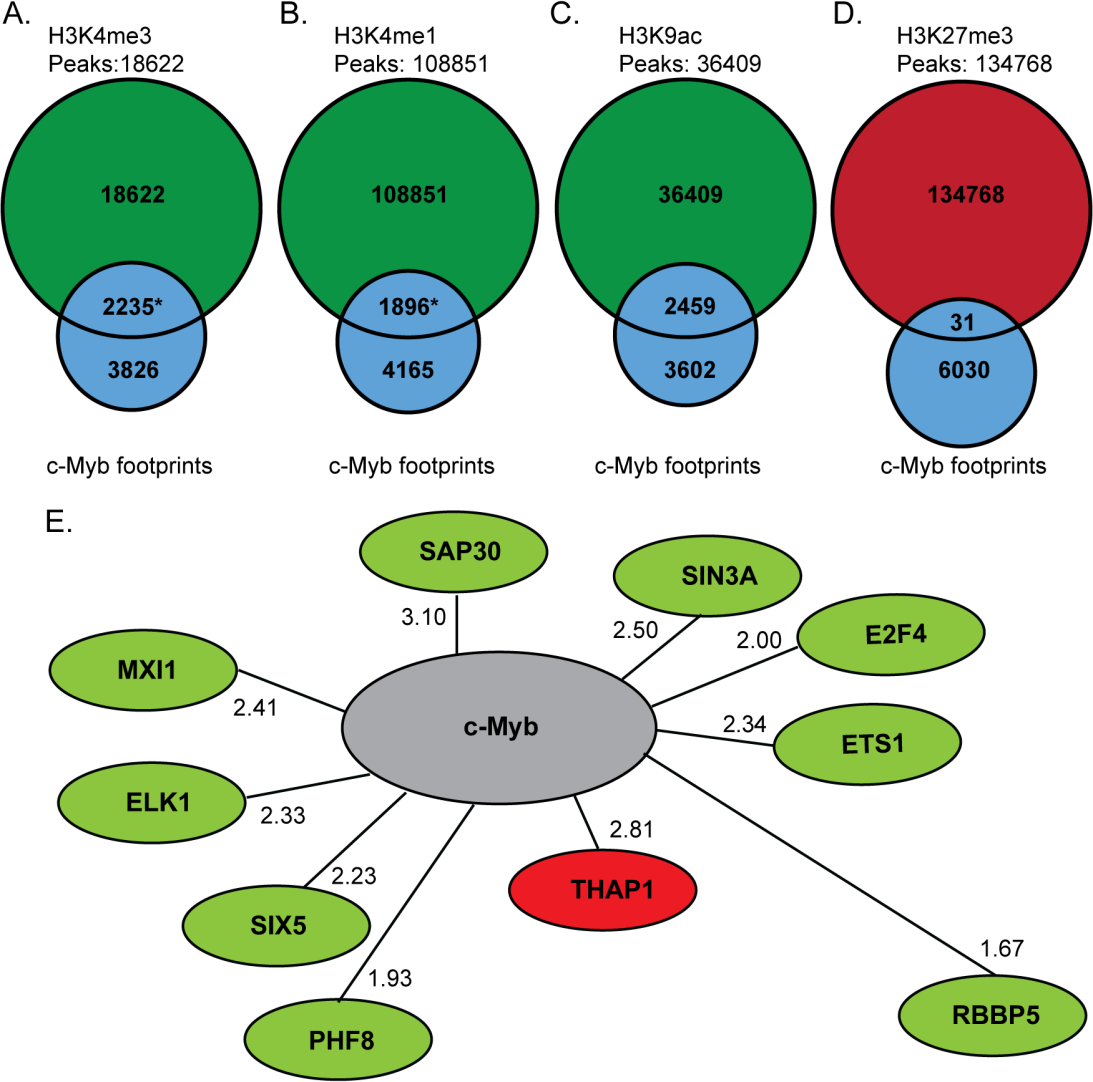
c-Myb footprints co-localises with active histone marks and co-regulatory TFs at c-Myb-regulated genes. (A-D) The number of c-Myb footprints (grey), which co-localize with ChIP-seq peaks for the active histone marks H3K4me3, H3K4me1, H3K9ac, (green) and the repressive mark H3K27me3 (red) in K562 cells. *Significantly different than expected from random sampling K562 DNase I footprints (p', > 4x10^-4^). (E) Suggested co-regulatory TFs for c-Myb in K562 cells. Green and red denotes factors in the positive and negative set respectively. Next to each factor, the normalised ratio for the co-localisation with c-Myb footprints at positively or negatively regulated genes is displayed. The distance between each factor and c-Myb is calculated as a measure of the normalized ratio, as described in Methods.

It has previously been reported that c-Myb acts as both a transcriptional activator and repressor and can influence the histone environment in the region it binds to [1,5,44,49]. To study how c-Myb footprints and histone marks correlate on a genome-wide level, we compared the identified c-Myb footprints to ChIP-seq peak datasets for four different histone marks (H3K4me3, H3K4me1, H3K9ac and H3K27me3) in K562 cells, available from the ENCODE Consortium (Farnham and Snyder labs) [50,51] (Fig 4A–4D). We found that 36.9% of the c-Myb footprints in K562 cells overlapped with ChIP-seq peaks of H3K4me3, a mark generally associated with transcriptional initiation (Fig 4A) [52,53]. This overlap represents 10.7% of total H3K4me3 peaks (1863 of 18622 peaks). Similar enrichments were found for H3K4me1 and H3K9ac, both marks associated with “open” chromatin and being signatures of enhancers [54]. Here we found an overlap of 31.3% of the c-Myb footprints with H3K4me1 peaks (Fig 4B) and 40.6% overlap of the c-Myb footprints with H3K9ac (Fig 4C). Only 1.7% of total ChIP peaks for H3K4me1 overlapped with c-Myb footprints. The repressive mark H3K27me3 [55–57] showed a very low overlap with only 31 (0.02%) c-Myb footprints falling inside 134768 H3K27me3 peaks (Fig 4D).

We next tested whether the overlap between c-Myb footprints and histone marks were different than expected by chance. We found that DNase I containing c-Myb footprints overlapped significantly with H3K4me3 peaks (positively, with r = 1.10) and H3K4me1 peaks (negatively, r = 0.81) from what is expected from a null model based on random sampling of DNase I footprints (p' < 4x10^-4^, Monte Carlo test) (S5A–S5D Fig and S6 Table). Furthermore, very few of a random sample of c-Myb motifs (same number as c-Myb footprints) overlapped with the different histone marks (S5E–S5H Fig). The general picture that emerges from this analysis is that c-Myb plays a role, both at enriched at TSS regions and exons, correlating with activating H3K4me3 marks. It also suggests that the repressive effects of bound c-Myb are achieved by other mechanisms than inducing repressive H3K27me3 marks.

The expression of a gene is often controlled by several TFs in concert through combinatorial control [58]. To obtain more information on how c-Myb exerts its function in synergy with other TFs in controlling gene expression of target genes, we analysed co-localisation of c-Myb footprints around the TSS and ChIP-seq peak datasets generated by the ENCODE Consortium [51] for 103 chromatin-associated proteins in K562 cells. We limited the analysis to the 467 genes positively or negatively regulated by c-Myb knockdown [1]. For each TF, we tested whether the ChIP-seq peaks overlapped c-Myb footprints around positively and negatively regulated genes, respectively, more than expected by random sampling of footprints. Based on certain thresholds (see Methods) we thus identified two sets of proteins that we suggest may co-regulate positively (9 factors) and negatively c-Myb regulated genes (1 factor), respectively (Fig 4E, S7 Table). Interestingly, c-Myb has previously been shown to interact with three of the proteins that we mapped to overlap on c-Myb target genes, either directly or as a part of complexes: a member of the mixed-lineage leukaemia (MLL) complex RBBP5 [49] and the two TFs ETS1 [59] and SIN3A [60]. Our analysis suggests that c-Myb may act together with these factors to modulate the expression of its target genes.

### c-Myb footprints are present on a subset of genes across six haematopoietic cell-types

**Fig 5:**
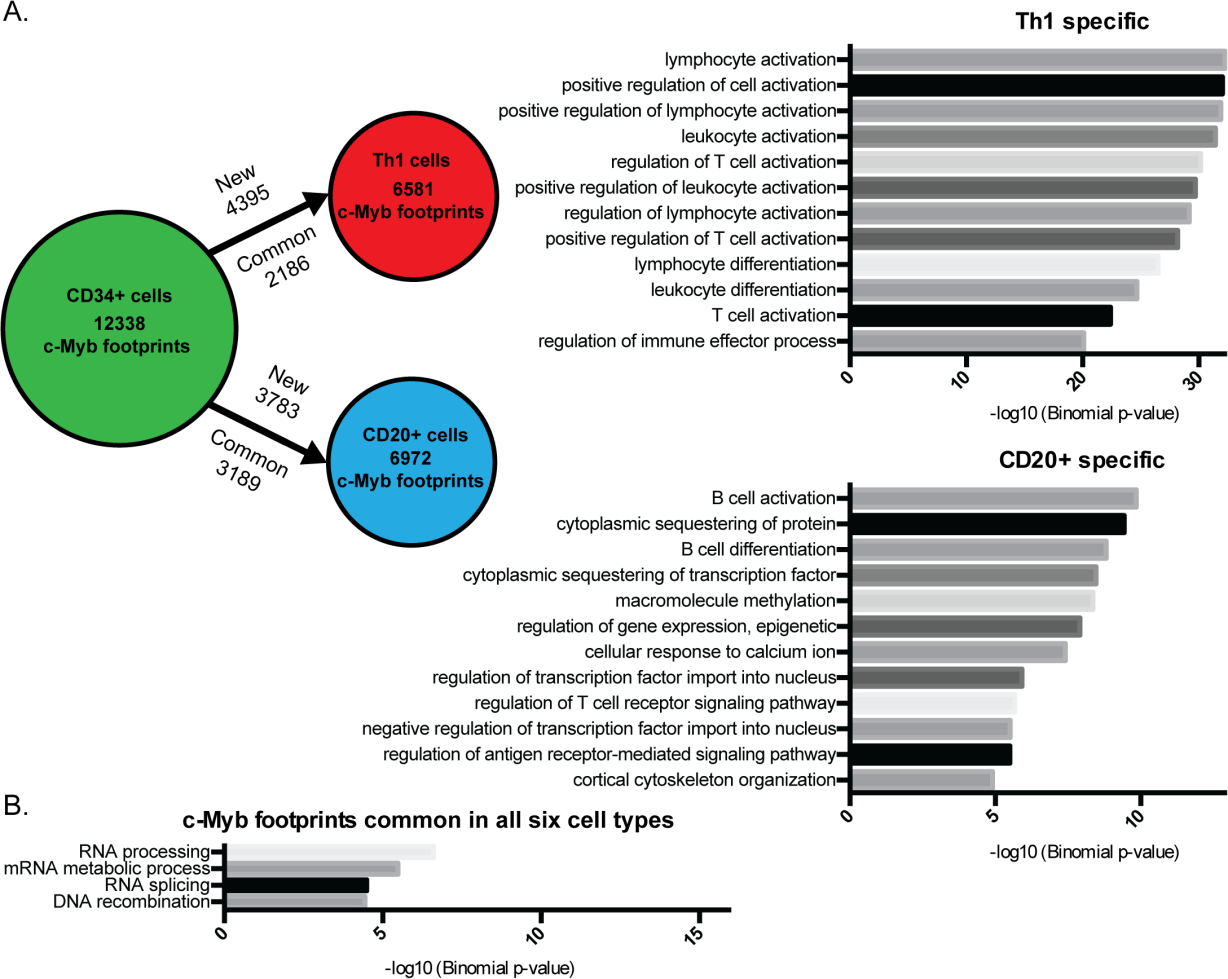
c-Myb controls differentiation and cell development. (A) Gain and loss of c-Myb footprints between CD34+ cells and CD20+ and Th1 cells, respectively. To the right are the top enriched functions for genes nearby c-Myb footprints specific for either CD20+ cells or Th1 cells as compared to CD34+ cells. For the full list of enriched functions, see S9 Fig. (B) Functional analysis of c-Myb footprints in all six cell-types. The functional analysis was made with GREAT [61].

To understand how c-Myb exerts its function through downstream gene programs, we assigned molecular functions to the identified c-Myb footprints through the use of the Gene Ontology (GO) tool GREAT [61,62] (Fig 5, S6–S9 Figs). For K562 cells the top enriched functions were identified to be in three groups: RNA catabolic processes, regulation of gene expression and cell cycle regulation (S6 Fig). This result correlates well with previous conclusions after c-Myb knockdown in the same cell type [1]. The functional analysis of the five other cell-types showed genes involved in cellular maintenance and several cell-specific functions were enriched for each cell type (S6–S9 Figs). We repeated the analysis for the same number of randomly selected DNase I footprints in all six cell-types and obtained results showing different gene functions from those predicted from the c-Myb footprint gene list.

To obtain more detailed information about the function of c-Myb in the different haematopoietic cells, we compared the c-Myb footprint genes from the haematopoietic progenitors CD34+ with c-Myb footprint genes from the more differentiated cell-types CD20+ and Th1 (Fig 5A). We found that a large number of c-Myb footprints are lost when the haematopoietic progenitors develop into each of the differentiated cell-types, while a small fraction of the c-Myb footprints is retained. However, an even larger fraction of the c-Myb footprints appear in this process and is specific for the differentiated cell type (Fig 5A). Functional analysis of the differentially mapped c-Myb footprint genes shows an enrichment of functions specific for the individual cell type, e.g. B cell activation and differentiation for CD20+ cells and T-cell activation and regulation for Th1 cells (Fig 5A).

A core of 406 common c-Myb footprints is present in all the six cell-types (e.g. *GRSF1*), and the functional analysis of this subset shows enrichment of genes involved in RNA processing and DNA recombination (Fig 5B, S10 Fig and S8 Table). We were concerned that these common c-Myb footprints could be driven by an overlap of DNase I footprints in all six cell-types and therefore we performed a random DNase I footprint control experiment ten times (S10 Fig). The random controls gave no common footprints, showing that there is a high degree of specificity for these common c- Myb footprints.

Four of the 65 common genes are listed among the genes regulated by c-Myb in K562 cells (*GRSF1*, *RUVBL2*, *UBE2N and SMNDC1*) (Figs 1G, 5B and S8 Table) [1]. Furthermore, when we analysed the list of common c-Myb footprints and compared overlap with ChIP-seq peaks for chromatin proteins that we identified as co-regulatory factors using c-Myb footprints in K562 cells (S7 and S9 Tables). A large fraction of the common c-Myb footprints (55–204) overlapped with ChIP-seq for the different factors.

To further validate our c-Myb footprints, we used the set of 406 common c-Myb footprints from our six cell-types and checked for overlap with c-Myb ChIP-seq peaks in human T-cell leukaemia cell lines (Jurkat and MOLT-3) [26]. The rationale is that if these footprints represent a common c-Myb signature, they should also be found among the c-Myb ChIP peaks in the two latter cell lines. We got an overlap of 65.2–75.8% in the Jurkat cell line and 79.6% in MOLT-3 the cell line. From this we can conclude that a large fraction of common c-Myb footprints from our analysis are also found in T-cell leukaemia cell lines. We illustrate an overlap of a common c-Myb footprint with the c-Myb ChIP-seq signal datasets at the GRSF1 promoter (S11A–S11B Fig).

## Discussion

In this study we have predicted genome-wide c-Myb binding in six different cell-types using digital DNase I footprints, from the haematopoietic progenitor CD34+ to the more differentiated cell-types GM12865, CD20+ and Th1 and the cancerous cell-types K562 and NB4 (Fig 1) [31]. Our aim was to evaluate whether DGF was an approach that could compensate for the lack of available c-Myb ChIP-seq data. With the filters utilised, we ended up with about 6000 footprints sharing a c-Myb signature in K562 cells. Several validation experiments suggested that these predictions had a reasonable accuracy. We used our c-Myb knockdown dataset from K562 cells to validate the c-Myb footprint predictions. For the top 100 c-Myb regulated targets a large proportion (39%) had c-Myb footprints +/- 10 kb from TSS, whereas when we extended the analysis to +/-100 kb, we detected c-Myb footprints at 72% of top 100 genes. Furthermore, we used reporter assays and showed that thirteen selected c-Myb footprint regions that localized either within the gene locus or upstream of twelve genes were enhanced to different degrees in the presence of c-Myb compared to control (Fig 2 and S3 Fig). In addition to these functional assays, we directly tested c-Myb occupancy on a selection of c-Myb footprints in K562 cells with the antibody independent technique DamID and showed that they indeed are elements recruiting c-Myb in their chromatin context (Fig 3 and S4 Fig). It is noteworthy that the level of c-Myb-Dam expression is very low in DamID compared to the reporter assay, and we were unable to detect the c-Myb-Dam fusion protein by western in c-Myb-Dam stable cell lines. That we nevertheless find c-Myb enriched in nine out of nine selected regions with c-Myb footprints suggests that c-Myb recognizes and selectively binds these predicted footprints in chromatin under quite stringent conditions. The DamID validations, therefore, lend quite a strong support to the accuracy of the DGF predictions.

The vertebrate Myb family members consist of A-Myb (*MYBL1*), B-Myb (*MYBL2*) and c-Myb (*MYB*) and share a conserved DNA-binding domain [63]. Although the Myb family members are very similar in overall structure and although they can be co-expressed in different cell-types, knockout studies of A-Myb, B-Myb or c-Myb show that they have differential roles in gene regulation during development and have distinct phenotypes [3,64,65]. The three MYB family members have their highest level of conservation in the DNA binding domain (DBD). They bind the same core Myb recognition element (MRE) (PyAACG/TG) [66–68] and the core MRE in c-Myb footprints in all six cell-types may therefore be bound by all three proteins (Fig 1D and S1G–S1K Fig). Our main focus has been on c-Myb footprints in K562 cells where c-Myb is the most highly expressed family member and overlapping binding of A-Myb to MREs is minimal, as *MYBL1* mRNA is approximately 900 times less abundant [1]. The *MYBL2* expression is four times lower than the expression of *MYB* in K562 cells and it is therefore a more likely candidate binder than *MYBL1* [1].While A- and c-Myb appear to have virtually identical DNA-binding properties, B-Myb forms complexes of significantly lower stability, which are rapidly dissociating under competitive conditions. It is therefore unlikely that B-Myb can form sufficiently stable enough complexes to generate clear DNase I footprints [69].

Another important aspect regarding prediction of specific TF footprint signatures is the residence time of the respective factor. A recent report by Hager and colleagues showed that DNase I “cleavage” signatures to a large extent depend on intrinsic properties of the DNase I and the DNA sequence in the factor-binding site [70]. However, the footprint depth seems to depend on the time the factor occupies and protects the target sequence. Many TFs with fast kinetics such as the glucocorticoid receptor (GR) gives poor overlap between GR footprints and ChIP-seq peaks compared to CTCF that has long residency time [70]. The *in vivo* dynamics of c-Myb binding is not known, but the intrinsic DNA binding properties of c-Myb has been extensively studied *in vitro*. Noteworthy, c-Myb binds to DNA in a two-step process—first the rapid formation of an unstable complex, followed by a slower transition to a stable complex, a process coupled with a conformational change in its DBD [71,72]. Therefore, c-Myb is expected to be able to bind more stably to chromatin than normal “tread milling” TFs. How this process is dependent on the DNA sequence in the factor-binding site remains to be elucidated.

Several methods for prediction of TF binding using DGF have been described in different cell-types from yeast to human [29–32,73–75]. Different computational prediction tools such as Wellington [75], CENTIPEDE [32], DNase2TF [70] and Footprint detection software [30] are available. We have devised an approach that uses DGF datasets from [31], in combination with MotifLab [37] and four c-Myb motifs from the TRANSFAC database [38] and weighted conservation using mammalian phastCons elements [41]. Our choice of conservation can be debated as regulatory elements may not necessarily be conserved across mammalian species [76]. A recent study showed that only about 22% of mouse TF footprints are conserved in human [77]. Even though several approaches have successfully identified active conserved regulatory regions across vertebrate species [78–81], many enhancers are poorly conserved and have species-specific TF binding [82,83]. Therefore, we cannot exclude the possibility that our filters will to a certain degree underestimate c-Myb binding sites in the six human cell-types. A recent report of an oncogenic super-enhancer formed by somatic mutation creating a novel c-Myb binding site shows that non-conserved enhancers can occur independently of evolution [26]. Our analysis, therefore, limits the prediction of c-Myb footprints to those that are evolutionarily conserved, and we may miss c-Myb regulatory elements only present in humans. We do, however, identify substantially more c-Myb footprints in our analysis as compared to the previously identified Myb footprints in seven lymphoblastoid cell lines [29].

Given these reservations, on a global level, our data show that c-Myb footprints differ from random sampling of DNase I footprints by locating more in exons than at promoters although total number of c-Myb footprints in promoters is higher in all cell-types. An estimate of 51% of all enhancers are intragenic [54] and DNaseI HS sites in exons have been implicated in chromatin looping and possibly alternative splicing [84].The presence of c-Myb in exons and a role in such processes is very interesting and needs to be further characterized in future studies.

We identified factors that co-localize with c-Myb footprints at promoters of c-Myb regulated genes in K562 cells [1] (Fig 4E). Three of the co-regulatory proteins (RBBP5, ETS1 and SIN3A) have been found to interact directly or indirectly with c-Myb [49,59,60]. SIN3A, SAP30 and RBBP5 are part of the ALL-1 super complex identified in K562 cells [85]. This complex also contains two other known c-Myb co-factors p300 [44] and CHD3 [86] that are involved in the regulation of c-Myb activity. Both p300 and CHD3 enhance c-Myb activity, and may function together with the SIN3A/SAP30/RBBP5 and c-Myb. RBBP5 is also part of the MLL1/2 complex responsible for H3K4me3 [87] and MLL3/4 was recently described as the methyltransferases that monomethylates H3K4 [88]. We find that one-third of c-Myb footprints overlapped with H3K4me3, and that this overlap was statistically significantly different than expected by random sampling of DNase I footprints (Fig 4A). MLL1 interacts with c-Myb through Menin [49] and, therefore, c-Myb may play a role in directing MLL mediated H3K4 trimethylation to c-Myb target genes.

Besides a small core of c-Myb footprints that are common across cell-types (total 406) (Fig 5B and S10 Fig), our analysis shows that a large part of c-Myb binding sites are cell type specific. Performing GREAT for the c-Myb footprints indicates that c-Myb has specialized roles related to the function of the specific cell type (Fig 5A and S6–S9 Figs).

The gene *GRSF1* is an important mitochondrial regulator and is one of the most affected genes upon c-Myb knockdown in K562 cells (S2 Table). Interestingly, our analysis identifies a c-Myb footprint in the promoter region of *GRSF1* present in all six cell-types. Moreover, we show that c-Myb is capable of enhancing the expression of *GRSF1* and also binds to the locus in K562 cells. Taken together, the data indicates that c-Myb is important for the expression of the *GRSF1* gene in several stages of the haematopoiesis.

We used this dataset of common c-Myb footprints and found extensive overlap with c-Myb ChIP-seq peaks in Jurkat and MOLT-3 cells, with the rationale is that if these footprints represent a common c-Myb signature, they should also be found among the c-Myb ChIP-peaks in the T-cell leukaemia cell lines. This was indeed true, we found a marked overlap that indicates that the common c-Myb footprints are bound by c-Myb, and may function as a type of quality control of our footprint predictions.

In summary, our data show that DGF can be used to predict conserved functional binding sites for c-Myb and that c-Myb has specific binding sites depending on the haematopoietic cell type. We have compared the majority of our analysis results to a random control. Furthermore, we have validated a selection of predicted c-Myb footprints by two different methods, and we found that c-Myb was capable of binding and enhancing gene activity through these predicted elements. We also mapped predicted c-Myb footprints to top c-Myb regulated target genes in K562 cells. These results suggest that a compelling fraction of our identified c-Myb footprints indeed are true c-Myb binding sites.

## Materials and Methods

### Data source

Digital genomic footprints for the six cell-types: CD20+, CD34+ (mobilized), GM12865, K562, NB4 and Th1 were obtained from [31]. ChIP-seq peaks for factors in K562 generated from experiments as part of the ENCODE Consortium [51] were downloaded from the UCSC Table Browser (S12 Table). For the histone analysis, we used ChIP-seq peaks generated by the Farnham and Snyder labs (S12 Table). For gene annotation data, we used ENSEMBL annotation GRCh37 [89].

### Cell culture

Human K562 cells and African green monkey CV1 cells were obtained from ATCC and cultured as described in [86].

### Constructs and Cloning

For luciferase constructs, genomic DNA was extracted from K562 cells using the DNeasy Blood & Tissue Kit (Qiagen). Selected genomic regions with the approximate size of 280 bp were amplified by PCR and cloned into the pGL.24.6 (Promega) vector using the restriction sites XhoI and NheI. For primers used, see S10 Table. To obtain the fusion construct 3xFLAG-c-Myb-V5-EcoDam, the c-Myb with an N-terminal 3xFLAG-tag was cloned into the pINDgw-RFA-V5-EcoDam using the Gateway technology (Invitrogen). The pINDgw RFA-V5-EcoDam, pIND-V5-EcoDam and pVgRXR vectors were a kind gift from Bas van Steensel [47]. c-Myb2KR is described in detail in [44]

### Reporter assay

The day before transfection, CV-1 cells were plated in 24 micro-well plates at 2x10^4^ cells per well. Cells were transfected with a total of 0.3 micrograms of DNA per well using the TransIT-LT transfection reagent (Mirus Bio). For the reporter assay 0.2 micrograms of pCIneo-c-Myb-2KR [1] and 0.1 micrograms of the pGL.4.26 were used per well. Cells were lysed 18 hours after post transfections with Passive lysis buffer (Promega) and luciferase activity was measured in a luminometer (Turner Designs). Data from three biological and nine independent transfections are presented.

### DNA adenine methyltransferase identification (DamID) assay

Stable K562 cell lines expressing either 3xFLAG-c-Myb-V5-EcoDam or EcoDam alone were generated by electroporation using the Amaxa Nucleofector system (Lonza Bioscience) with the pINDgw-3xFLAG-c-Myb-V5-EcoDam or pIND-V5-EcoDam, respectively. Following electroporation cell lines were selected with G418 (Invivogen). DamID libraries for EcoDam and c-Myb-V5-EcoDam were made as described in [47]. In brief: Genomic DNA was isolated using the DNeasy Blood & Tissue Kit (Qiagen) and processed to enrich for DNA methylated by either V5-EcoDam alone or 3xFLAG-c-Myb-V5-EcoDam. Purified DNA was analysed by qPCR using the same amount of DNA for EcoDam and c-Myb-V5-EcoDam [48] on a Lightcycler480 (Roche). For primers used, see S10 Table. To validate the expression of the full-length 3xFLAG-c-Myb-V5-EcoDam construct, K562 cells were transfected with pINDgw-3xflg-c-Myb-V5-EcoDam or pIND-V5-EcoDam respectively together with the pVgRXR ecdysone receptor-encoding vector. Next day, 2 μM of Ponasterone A (Invitrogen) was added to the cell media and after 24 hours cells were lysed in SDS loading dye and subjected to western blotting on a PVDF membrane with anti-FLAG (Sigma) and anti-GAPDH (Invitrogen) antibodies (S10 Table).

### Identification of c-Myb footprints

To predict c-Myb footprints in the human genome (hg19), we used the MotifLab analysis workbench with MATCH motif scanning tool and *minSUM* cut-off threshold [37,90]. We scanned with four c-Myb motif models (M00004, M00183, M00773, M00913) from the TRANSFAC database [38]. The overlap between the c-Myb motif instances and DNase I footprints was calculated for each of the six cell-types with a threshold of 0.9 (CD20+, CD34+ (mobilized), GM12865, K562, NB4 and Th1) [31]. For each predicted motif instance we calculated a weighted average conservation score across the site where the conservation score (phastCons46wayPlacental) (http://hgdownload.cse.ucsc.edu/goldenPath/hg19/phastCons46way/placentalMammals/) in each position was weighted according to the information content of the corresponding column in the motif. Sites that scored below 0.22 were discarded from further consideration. The *de novo* search for motifs inside the c-Myb footprints was carried out with the motif identification tool ChIPMunk [42].

### c-Myb regulated gene set

The list of 100 most up- or down-regulated genes upon c-Myb knockdown in K562 cells are obtained from [1]. In brief, we analyzed the global effects of c-Myb knockdown using microarray expression profiling by comparing genome wide patterns of gene expression between control and c-Myb-siRNA transfected K562 cells. The control K562 cells were transfected with a non-specific siRNA (siLuc; targeting the firefly luciferase gene). We performed a first profiling experiment using eight biological replicates and si323 RNA-mediated knockdown. A second expression profiling study with the si2992 RNA-mediated knockdown and four biological replicates was used to validate the regulated genes detected in the first dataset. After statistical analysis of the results from each of the experiments using permutation F2-tests, in which residuals were shuffled 5000 times, and family-wise error correction, and top 100 significantly regulated genes (P<0.05) were selected.

### Statistical analysis

For statistical analysis we used The Genomic HyperBrowser [91]. Hypothesis testing was performed using Monte Carlo simulation with 10000 repetitions, drawing random samples (of the same size as the number of c-Myb footprints) uniformly from the total population of DNase I footprints. As the test statistic, the difference in the overlap between the dataset in question and respectively the sampled footprints (case) and the rest (control) was used. The p-values were corrected for multiple testing using FDR correction over all tests, or in the case of the analysis of the cell-specific distribution of c-Myb footprints, over all tests per cell type [92]. As a measure of effect size, a normalised overlap ratio was used, defined as follows:

r=(Xm/Yn)

where *X* is the overlap between the query dataset and c-Myb footprints, *Y* is overlap between the query dataset and the rest of the DNase I footprints, *n* is the number of c-Myb footprints and *m* is the number of remaining DNase I footprints. For these analyses the middle point of the DNase I footprints were used.

### Analysis of TF co-regulation

For the analysis of TF co-regulation, distance from c-Myb footprints to the closest gene regulated by c-Myb [1] was assigned using BEDOPS [93]. Footprints inside +/- 5 kb of TSS of regulated genes were isolated and compared with ChIP-seq datasets. Several thresholds were set: first, only factors with peaks overlapping the gene-regulating c-Myb footprints significantly more than expected by random sampling of DNase I footprints, were selected (FDR-corrected p-value, p', < 0.05); second, the threshold for normalised ratio was set to 1.05; third, there needed to be at least 20 genes with c-Myb regulating footprints (both positively and negatively) overlapping the ChIP-seq peaks of the factor; and fourth, the difference in normalised ratio for the overlap between the peaks and the positively and negatively regulated genes, respectively, needed to be > 0.5. Factors thus selected were then assigned to either a positive or a negative set of co-regulating TFs according to the highest value of the normalised ratio. A distance measure between c-Myb and each protein was calculated as:

$$10^{\frac{a}{b}}$$

Where *a* is the highest normalised ratio for the factors in the set, and *b* is the normalised ratio of the factor in question.

### Distribution of c-Myb footprints

To calculate the genomic distribution of the c-Myb footprints, Ensembl gene annotations were used. The annotations were divided into the following categories: promoters, exons, 3´-UTR, introns, and intergenic regions. The promoter regions were defined as -2500 bp upstream and 500 bp downstream of TSS. In cases where a footprint was found in more than one gene category, it was assigned to one category in the following order: promoters, exons, 3´-UTR, introns, and intergenic regions. For the distribution around TSS, c-Myb footprints, DNase I footprints and c-Myb motifs were grouped into 100 bp bins and summed. For all analyses, including histone marks and distance to regulated genes, the middle point of the DNase I footprints and motifs were used.

### GREAT analysis

For the functional analysis of c-Myb footprints the GREAT tool with standard settings was used [61]. The middle point of either c-Myb footprints or a random selection containing the same number of cell- specific DNase I footprints was used as input. For a comparison of c-Myb specific footprints between cell-types, the middle point of c-Myb specific footprints were expanded with 12 bp on each side and an overlap between two footprints was set to require at least six bp. The promoter regions of the gene lists are defined as -2.5 kb upstream to +0.5 kb downstream of the TSS.

### Analysis of c-Myb ChIP-seq data

For analysis of c-Myb ChIP-Seq data from [26], datasets were retrieved from NCBI Gene Expression Omnibus (GEO) (GSM1519643 and GSM1442006) and analysed with SraTailor [94] using the programs standard settings for Bowtie2 [95] and MACS [96]. ChIP-seq datasets for c-Myb were analysed for enrichment with corresponding control datasets. To calculate the fraction of common footprints in all six cell-types co-localising with ChIP-Seq peaks for c-Myb in Jurkat and MOLT-3 cells, the overlap between footprint and peaks was set to be a minimum of one bp.

## Supporting Information

S1 Fig Weighted average conservation for genome-wide c-Myb motifs and c-Myb footprints. (A-F) Weighted average conservation using mammalian phastCons elements for each predicted motif instance for all genome-wide were calculated for c-Myb motifs and the identified c-Myb footprints +/- SD, respectively, in the cell-types CD20+, CD34+, GM12865, K562, NB4, Th1. (G-I) The binding motif enriched in c-Myb footprints in CD20+, CD34+, GM12865, NB4 and Th1 cells.
(TIF)

S2 Fig Genomic distribution of c-Myb footprints. (A-E) Localization of c-Myb footprints, a random selection of DNase I footprints and a random selection of c-Myb motifs for the cell-types CD20+, CD34+, GM12865, NB4 and Th1 around TSS. (F) Genomic distribution of c-Myb footprints for the cell-types CD20+, CD34+, GM12865, NB4 and Th1. *Overlapping significantly more with c-Myb footprints than with randomly selected K562 DNase I footprints (p' < 0.05, calculated by the Monte Carlo test).
(TIF)

S3 Fig Additional luciferase assays. (A-H) Luciferase assay as described in Fig 2.
(TIF)

S4 Fig Additional DamID assays. (A) Schematic overview of the DamID method. (B-D) DamID assay for the association of the control Dam and c-Myb-Dam as described in Fig 3.
(TIF)

S5 Fig Co-localisation of DNase I and c-Myb motifs with histone marks. (A-H) Overlap between ChIP-seq peaks for the active histone marks H3K4me3, H3K4me1, H3K9ac (green) and the repressive mark H3K27me3 (red) in K562 cells, and K562 DNase I footprints or a random sample of c-Myb motifs. For DNase I footprints, the expected number of overlapping footprints when drawing random samples without replacement from the total set of K562 DNase I footprints (the hypergeometric distribution) are shown. For c-Myb motifs the overlaps of a single random sample are shown.
(TIF)

S6 Fig Functional analysis of c-Myb footprints in K562 cells and CD20+ cells. GREAT GO-term annotations for c-Myb footprints and a random sample of DNase I footprints for K562 cells (A-B) and CD20+ cells (C-D).
(TIF)

S7 Fig Functional analysis of c-Myb footprints in CD34+ cells and GM12865 cells. GREAT GO-term annotations for c-Myb footprints and a random sample of DNase I footprints for CD34+ cells (A-B) and GM12865 cells (C-D).
(TIF)

S8 Fig Functional analysis of c-Myb footprints in NB4 cells and Th1 cells. GREAT GO-term annotations for c-Myb footprints and a random sample of DNase I footprints for NB4 cells (A-B) and Th1 cells (C-D).
(TIF)

S9 Fig Functional analysis of cell-specific c-Myb footprints in CD20+ cells and Th1 cells. The full list of enriched functions identified with GREAT for cell specific c-Myb footprints for CD20+ cells (A) and Th1 cells (B) as compared to CD34+ cells.
(TIF)

S10 Fig Analysis of overlap of c-Myb footprints in the six cell-types compared to random DNase I footprint controls. Graphs showing number of common c-Myb footprints or random selections of cell-specific DNase I footprints after subtraction of non-overlapping footprints between two cell-types at the time, and ending with the final number is a common set of footprints in all six cell-types. The analysis of a random selection of cell-specific DNase I footprints was repeated ten times starting with 12338 random footprints in CD34+ cells. The y-axis represents the number of c-Myb or DNase I footprints; the x-axis shows the six cell-types with total number of c-Myb footprints or number of random selection of cell-specific DNase I footprint used in the analysis (c-Myb footprints, red graph; random DNase I footprints, black bars). The numbers to the right indicate common footprints for c-Myb (red) or a random selection of cell-specific DNase I footprints (black) footprints common in all the cell-types.
(TIF)

S11 Fig Overlap of common c-Myb footprints and c-Myb ChIP-Seq data from Jurkat and MOLT-3 cells. A) Overlap between c-Myb ChIP-Seq peaks for Jurkat and MOLT-3 cells [26] and the c-Myb footprints common in all the six cell-types analysed in this study. ChIP-Seq data was processed with SraTailor [94] using the default settings. B) An illustration showing the identified c-Myb common footprints at the promoter for GRSF1 for the six cell-types analysed in this study (see also Fig 1F) and enriched c-Myb ChIP-Seq signals for the same region in Jurkat and MOLT-3 cells. Coordinates for c-Myb footprint are shown above, and to the left are the signal intensities for the ChIP-Seq data shown. UCSC version hg19 (http://genome.ucsc.edu).
(TIFF)

S1 Table DNase I footprints and c-Myb footprints for the six cells types analysed. The total number of footprints, footprints overlapping with c-Myb motifs and predicted c-Myb footprints in all the six cell-types analysed.
(PDF)

S2 Table The ten most downregulated genes in K562 cells upon knockdown of c-Myb. Gene name, ID number, degree of regulation, if the gene contains a c-Myb footprint and the position of the footprint for the ten most downregulated genes in K562 cells upon knockdown of c-Myb [1].
(PDF)

S3 Table The ten most upregulated genes in K562 cells upon knockdown of c-Myb. Gene name, ID number, degree of regulation, if the gene contains a c-Myb footprint and the position of the footprint for the ten most upregulated genes in K562 cells upon knockdown of c-Myb [1].
(PDF)

S4 Table Genomic localisation for c-Myb footprints in the six cell-types analysed. FDR-corrected p-values, p', and normalised ratio for the distribution of c-Myb footprints at annotated genes, promoters and intergenic regions for the six cell-types analysed.
(PDF)

S5 Table Additional c-Myb target genes used in this study. Additional genes used in the study that are also regulated in K562 cells upon knockdown of c-Myb [1].
(PDF)

S6 Table Co-localisation between c-Myb footprints and histone modifications. FDR-corrected p-values, p', and normalised ratios for co-localisation between c-Myb footprints and ChIP-Seq peaks for the four histone modifications analysed in K562 cells.
(PDF)

S7 Table Co-regulatory transcription factors. Transcription factors with ChIP-seq peaks significantly overlapping c-Myb footprints at positively and negatively c-Myb-regulated genes. FDR-adjusted p-values, p', and normalised ratios are shown. For full list of ChIP-seq datasets analysed see S12 Table.
(PDF)

S8 Table Genes enriched in the GO terms of c-Myb footprints common in all six cell-types. Gene list enriched in the four GO-terms (Fig 5B) identified by GREAT for c-Myb footprints common in all the six cell-types analysed in this study. The promoter regions of the genes are defined as -2.5 kb upstream to +0.5 kb downstream of the TSS.
(PDF)

S9 Table Overlap between suggested co-regulatory factors in K562 cells and c-Myb footprints common in all six cell-types. (PDF)

S10 Table List of primers used in this study. (PDF)

S11 Table Antibodies used in this study. (PDF)

S12 Table List of ChIP-Seq peak datasets used in this study. (PDF)

S1 File c-Myb footprints identified in the six cell-types analysed. (ZIP)
