# Supplementary figures and images for: c-Myb Binding Sites in Haematopoietic Chromatin Landscapes
Source: PLoS One. 2015 Jul 24;10(7):e0133280. doi: 10.1371/journal.pone.0133280 (PMC4514710; doi:10.1371/journal.pone.0133280)

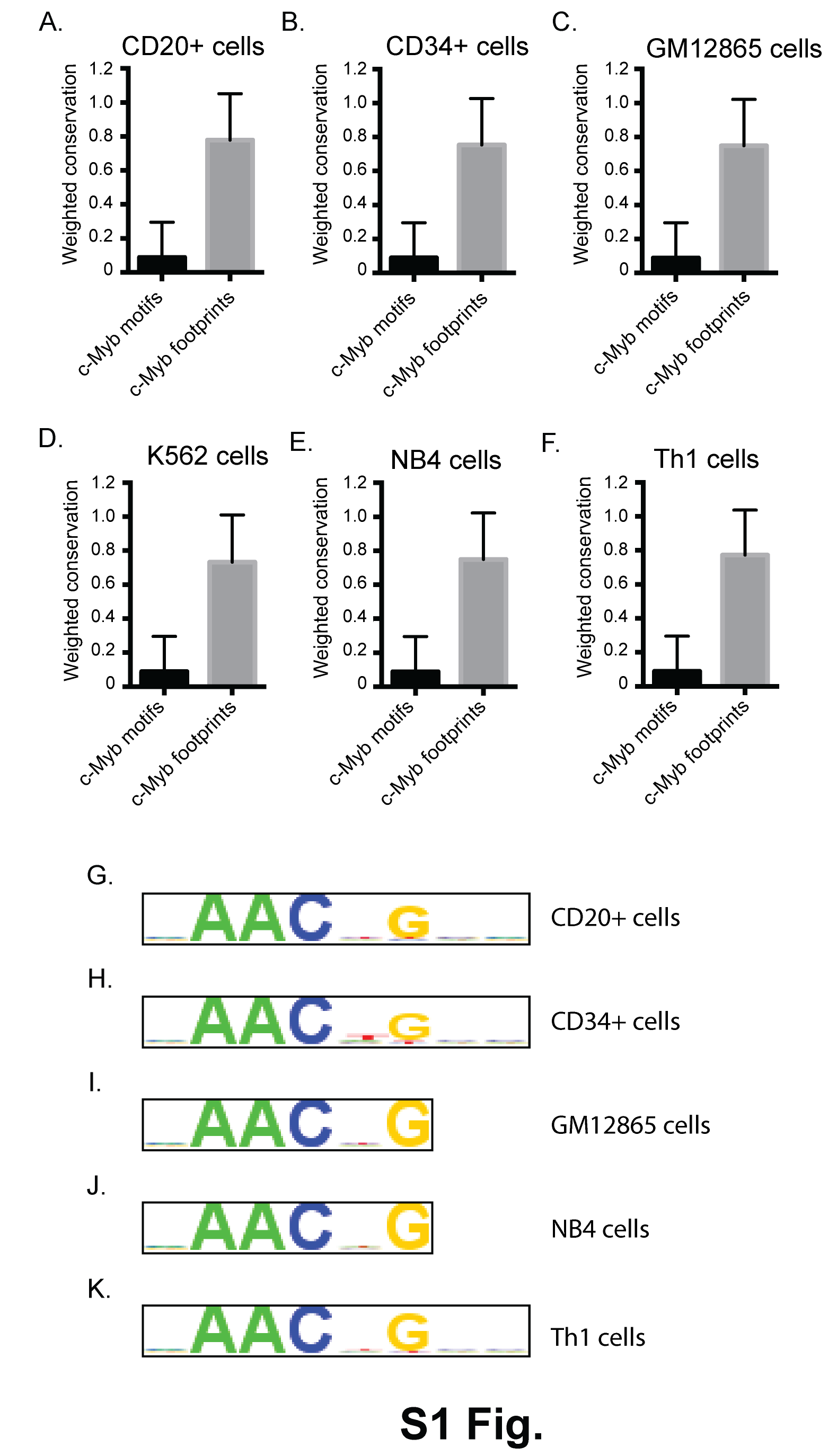

Supplement: S1 Fig — (A-F) Weighted average conservation using mammalian phastCons elements for each predicted motif instance for all genome-wide were calculated for c-Myb motifs and the identified c-Myb footprints +/- SD, respectively, in the cell-types CD20+, CD34+, GM12865, K562, NB4, Th1. (G-I) The binding motif enriched in c-Myb footprints in CD20+, CD34+, GM12865, NB4 and Th1 cells. (TIF) [file pone.0133280.s001.tif]

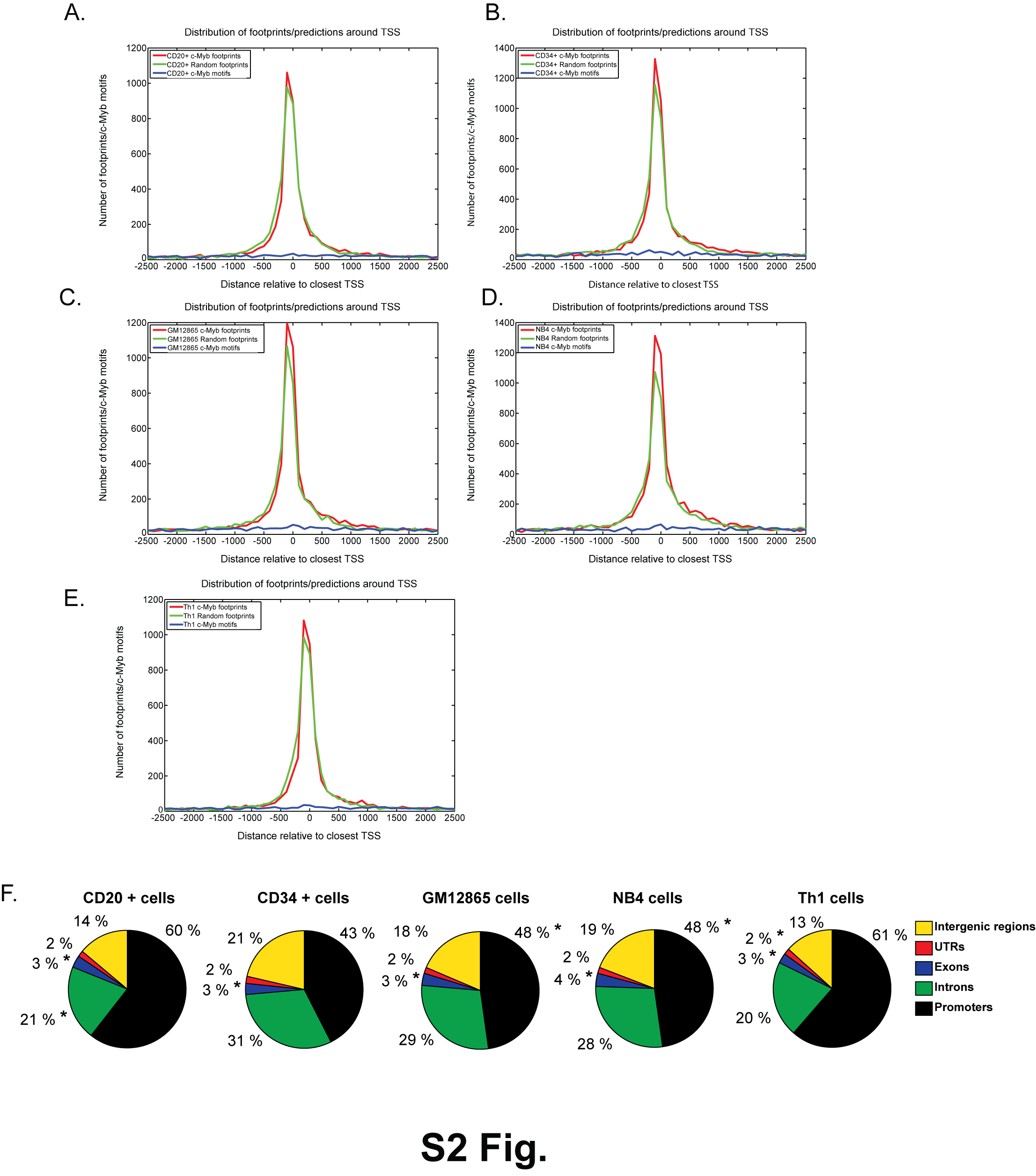

Supplement: S2 Fig — (A-E) Localization of c-Myb footprints, a random selection of DNase I footprints and a random selection of c-Myb motifs for the cell-types CD20+, CD34+, GM12865, NB4 and Th1 around TSS. (F) Genomic distribution of c-Myb footprints for the cell-types CD20+, CD34+, GM12865, NB4 and Th1. *Overlapping significantly more with c-Myb footprints than with randomly selected K562 DNase I footprints (p' < 0.05, calculated by the Monte Carlo test). (TIF) [file pone.0133280.s002.tif]

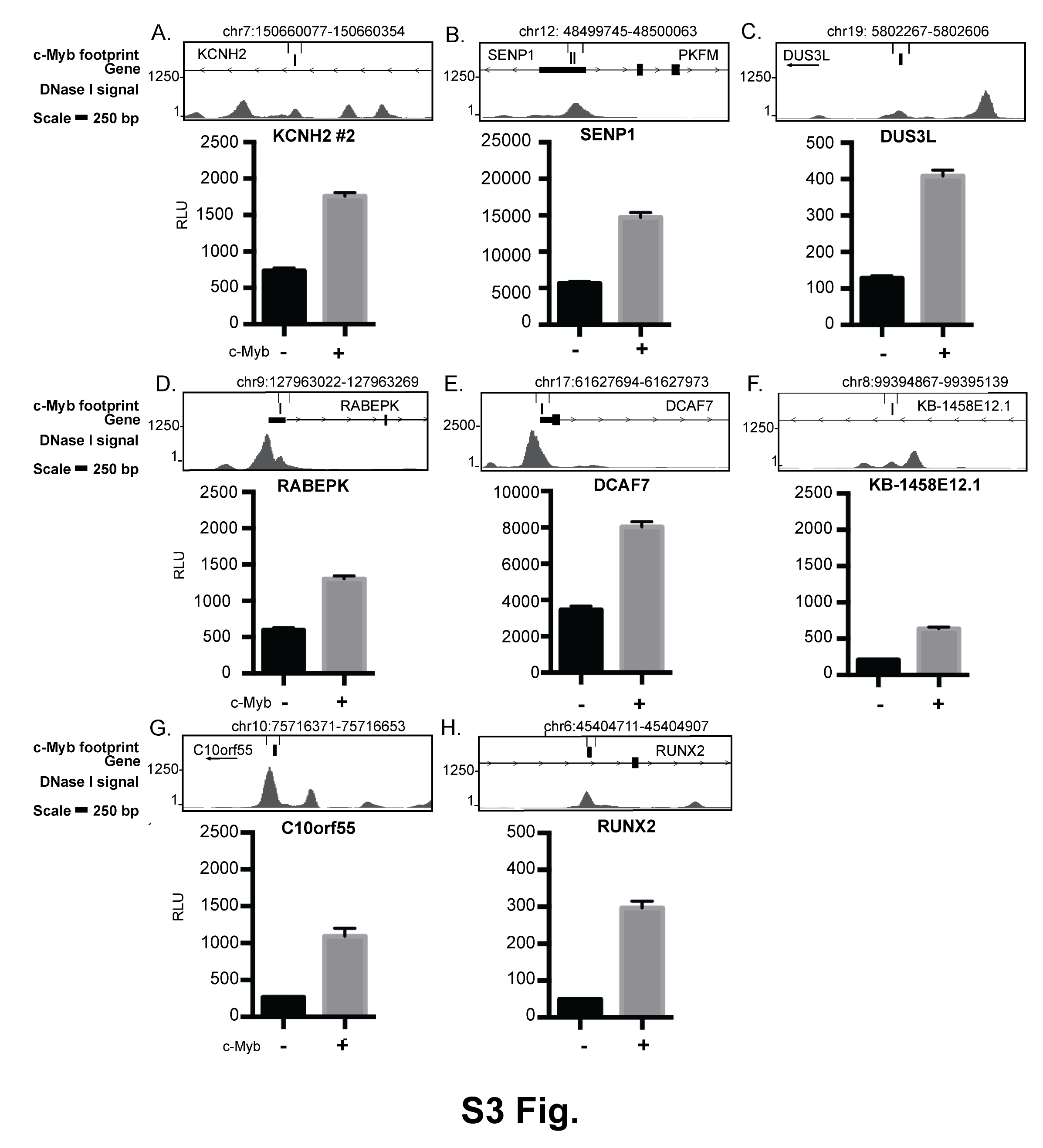

Supplement: S3 Fig — (A-H) Luciferase assay as described in Fig 2. (TIF) [file pone.0133280.s003.tif]

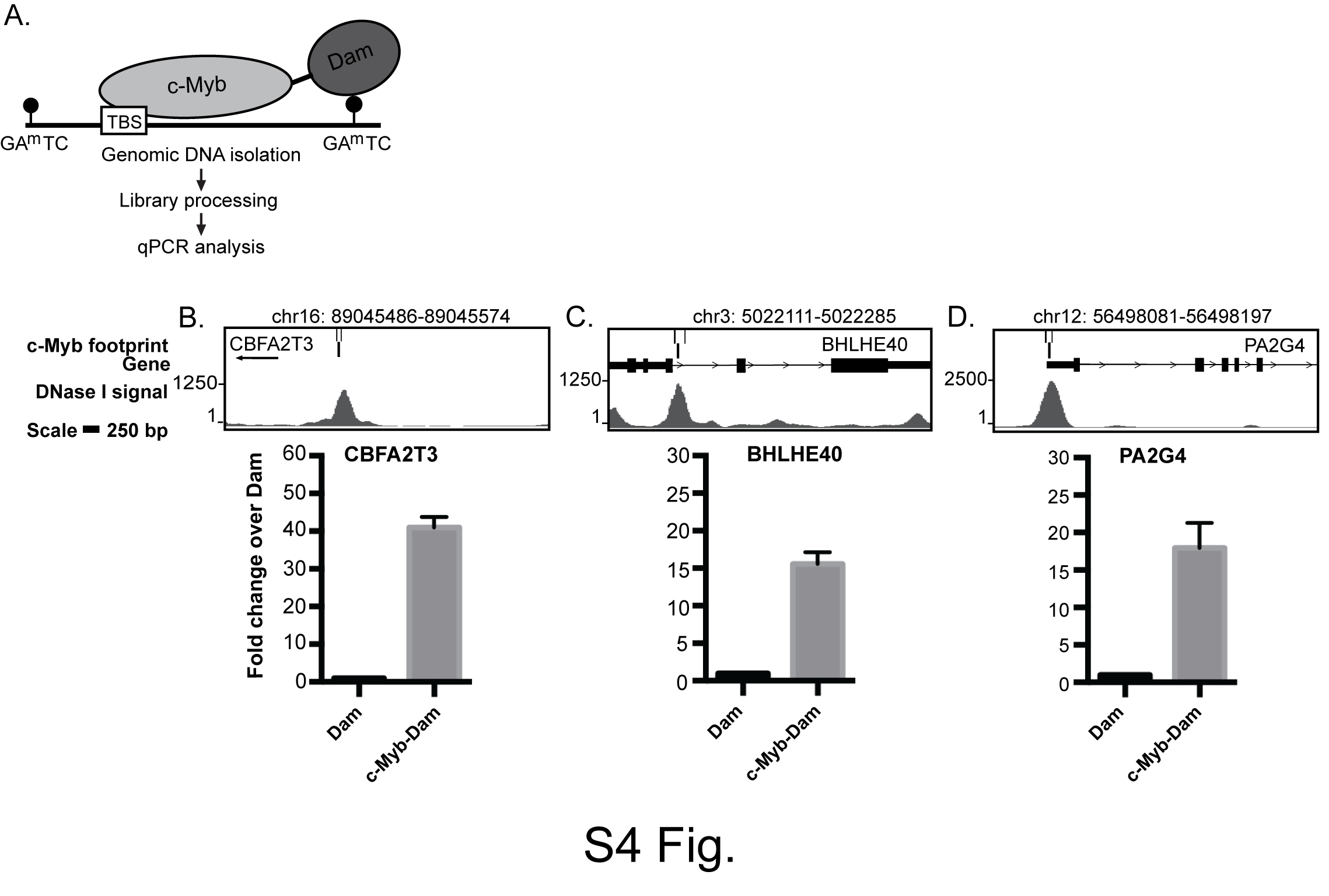

Supplement: S4 Fig — (A) Schematic overview of the DamID method. (B-D) DamID assay for the association of the control Dam and c-Myb-Dam as described in Fig 3. (TIF) [file pone.0133280.s004.tif]

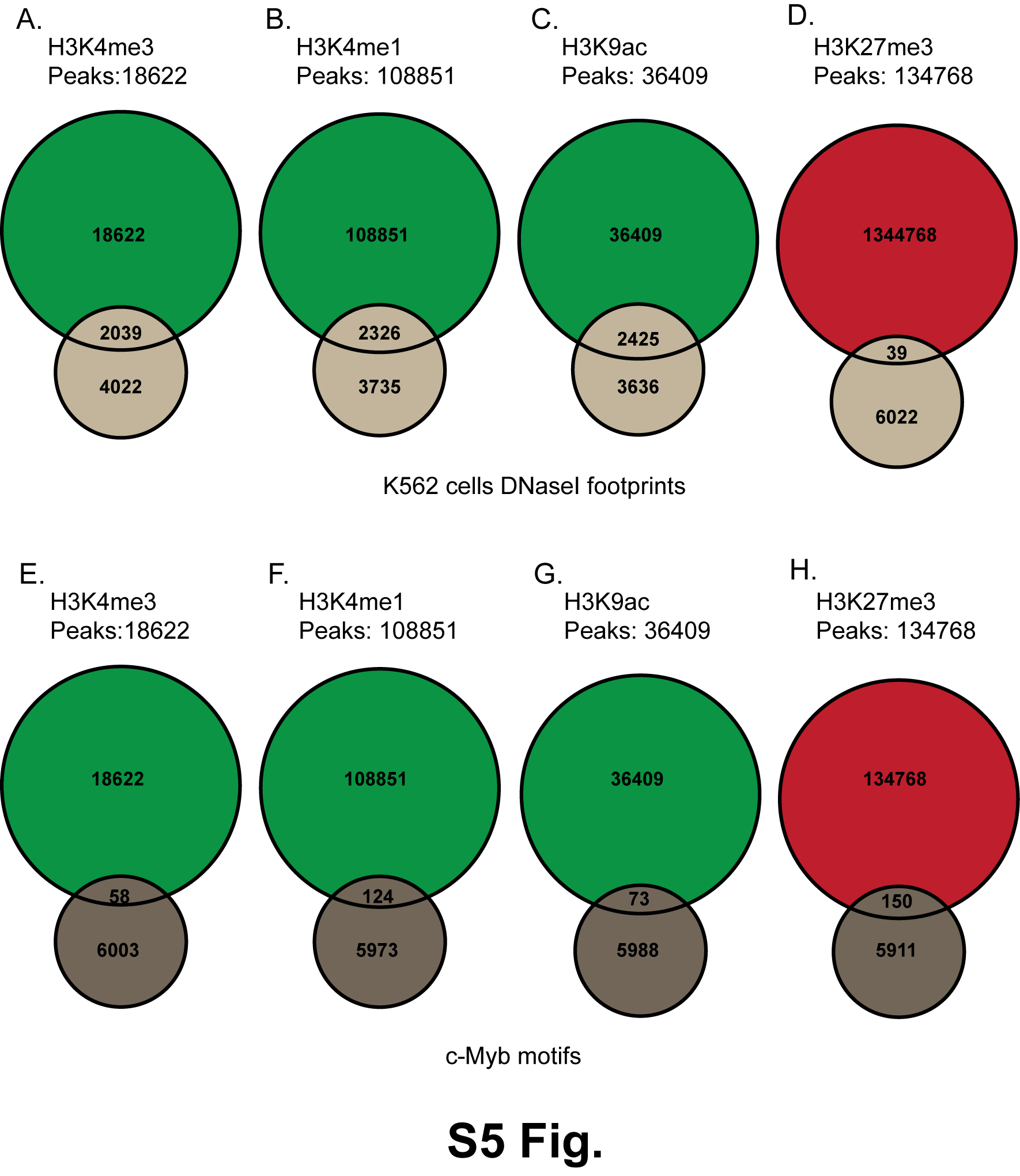

Supplement: S5 Fig — (A-H) Overlap between ChIP-seq peaks for the active histone marks H3K4me3, H3K4me1, H3K9ac (green) and the repressive mark H3K27me3 (red) in K562 cells, and K562 DNase I footprints or a random sample of c-Myb motifs. For DNase I footprints, the expected number of overlapping footprints when drawing random samples without replacement from the total set of K562 DNase I footprints (the hypergeometric distribution) are shown. For c-Myb motifs the overlaps of a single random sample are shown. (TIF) [file pone.0133280.s005.tif]

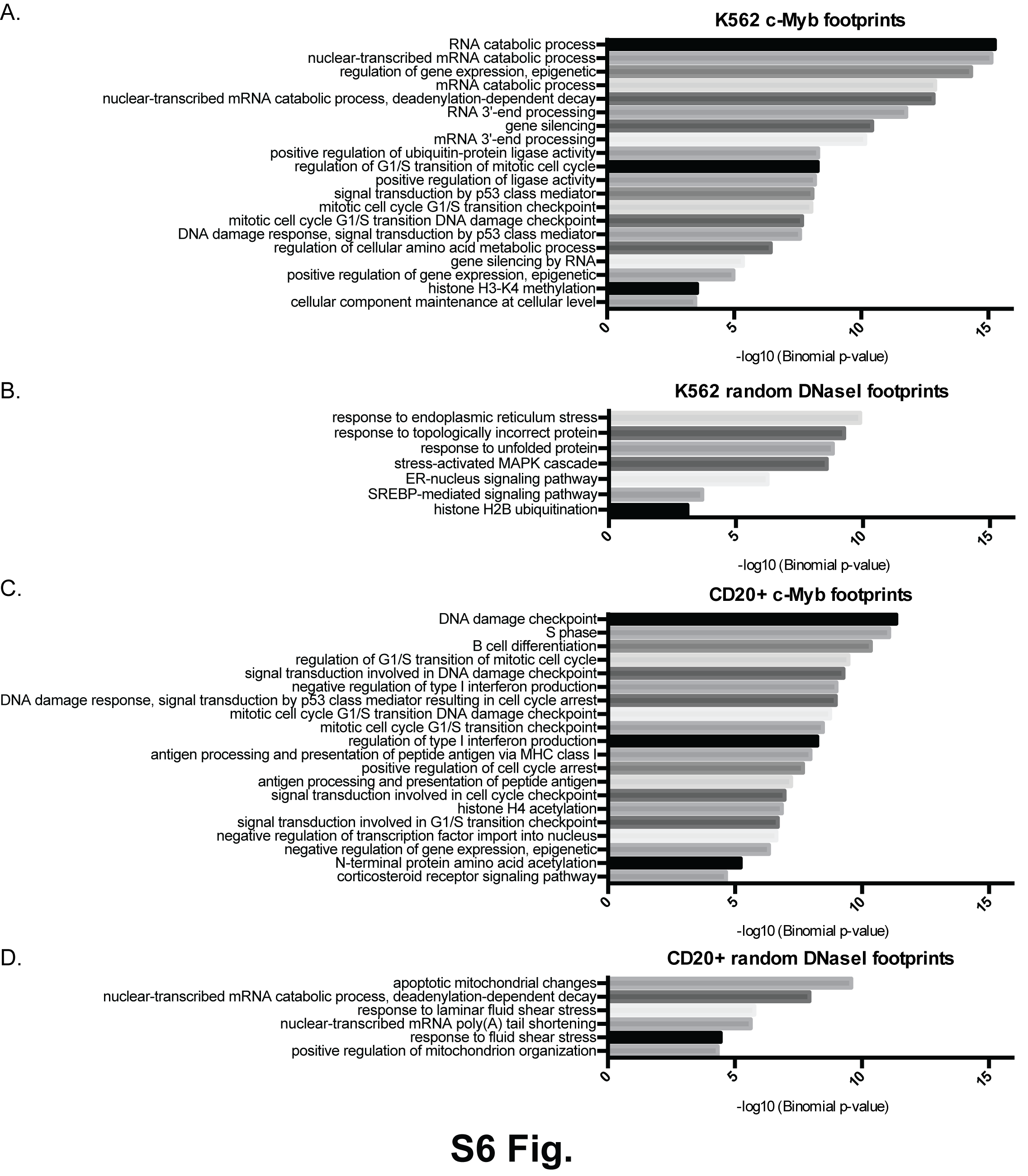

Supplement: S6 Fig — GREAT GO-term annotations for c-Myb footprints and a random sample of DNase I footprints for K562 cells (A-B) and CD20+ cells (C-D). (TIF) [file pone.0133280.s006.tif]

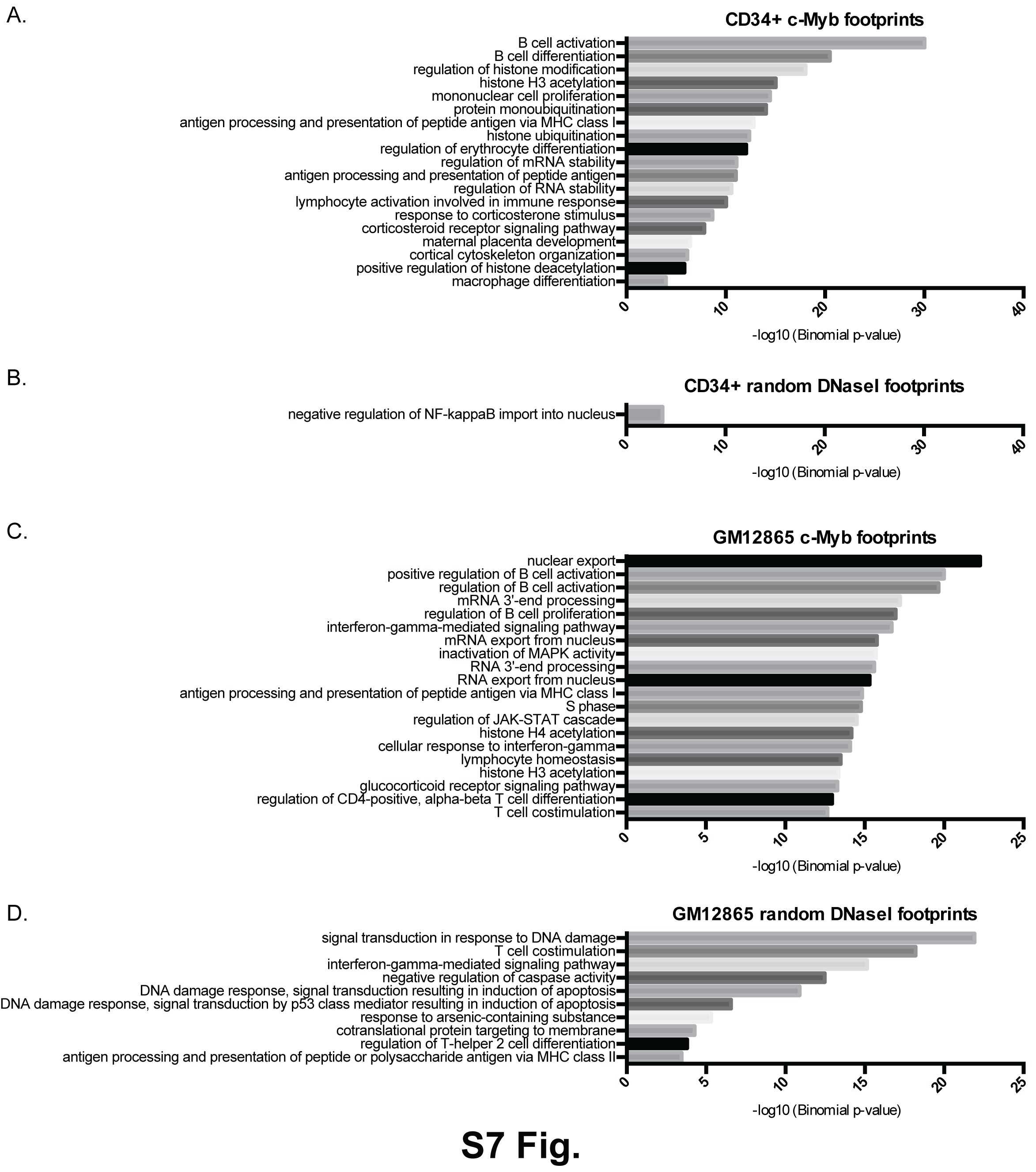

Supplement: S7 Fig — GREAT GO-term annotations for c-Myb footprints and a random sample of DNase I footprints for CD34+ cells (A-B) and GM12865 cells (C-D). (TIF) [file pone.0133280.s007.tif]

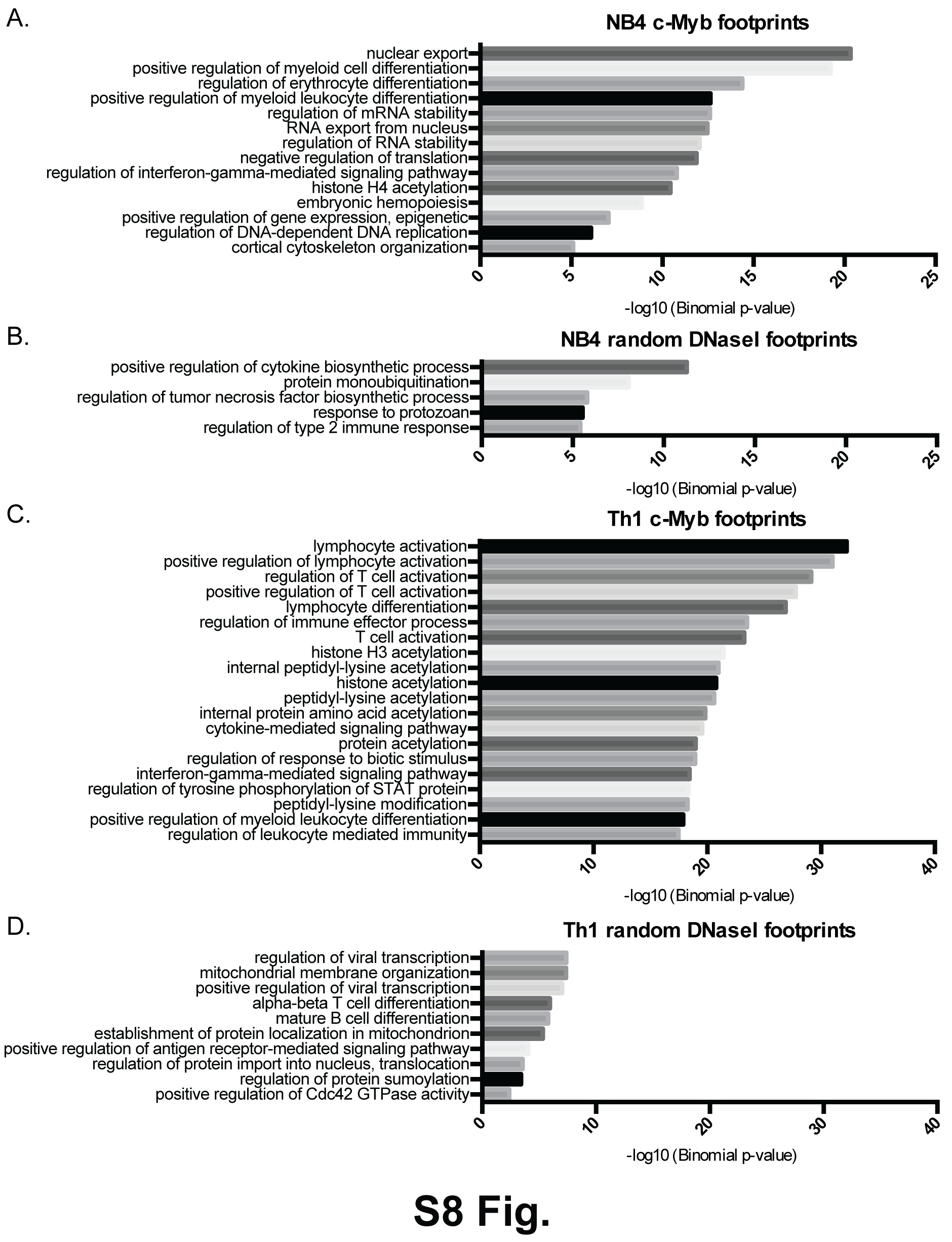

Supplement: S8 Fig — GREAT GO-term annotations for c-Myb footprints and a random sample of DNase I footprints for NB4 cells (A-B) and Th1 cells (C-D). (TIF) [file pone.0133280.s008.tif]

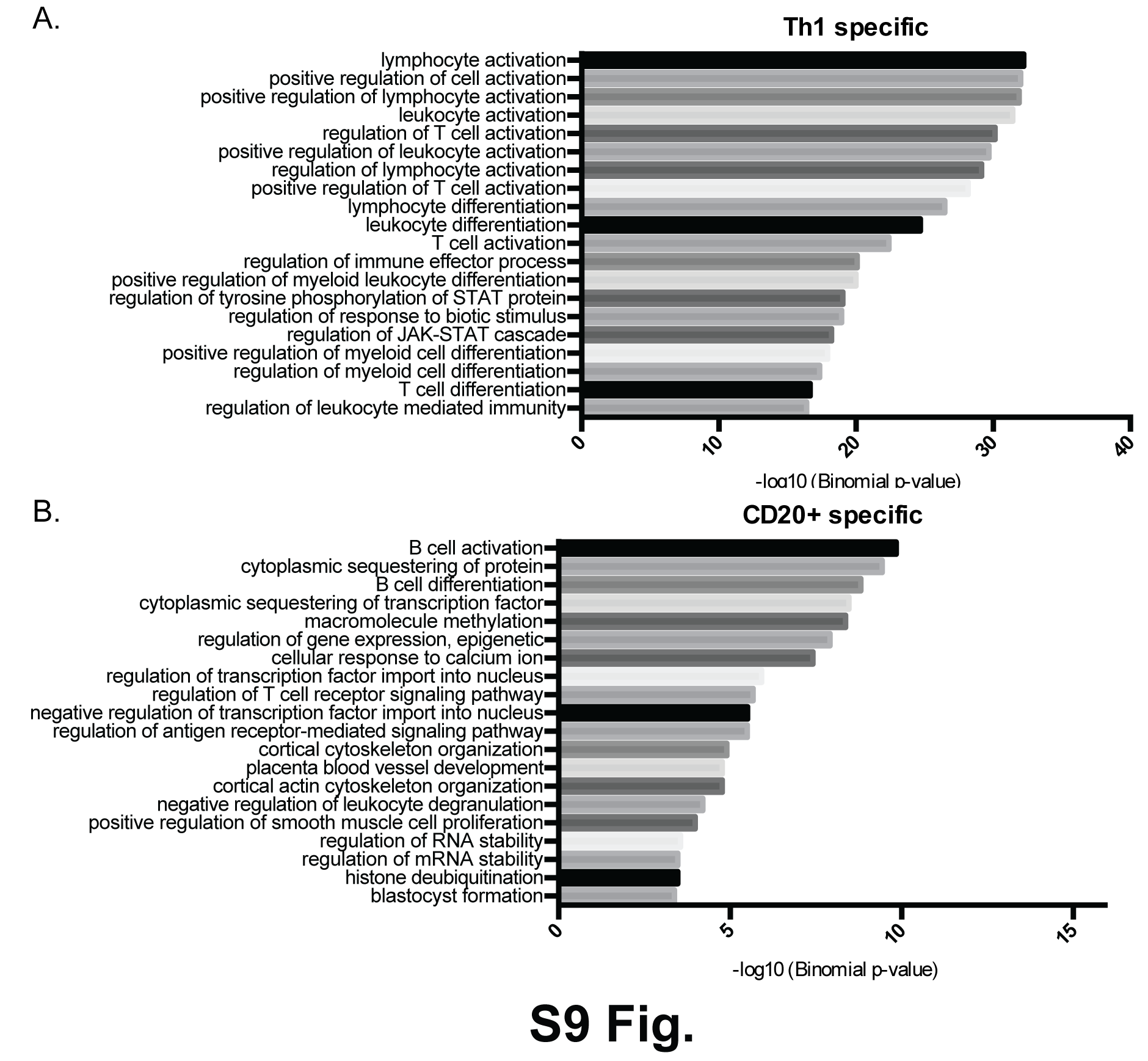

Supplement: S9 Fig — The full list of enriched functions identified with GREAT for cell specific c-Myb footprints for CD20+ cells (A) and Th1 cells (B) as compared to CD34+ cells. (TIF) [file pone.0133280.s009.tif]

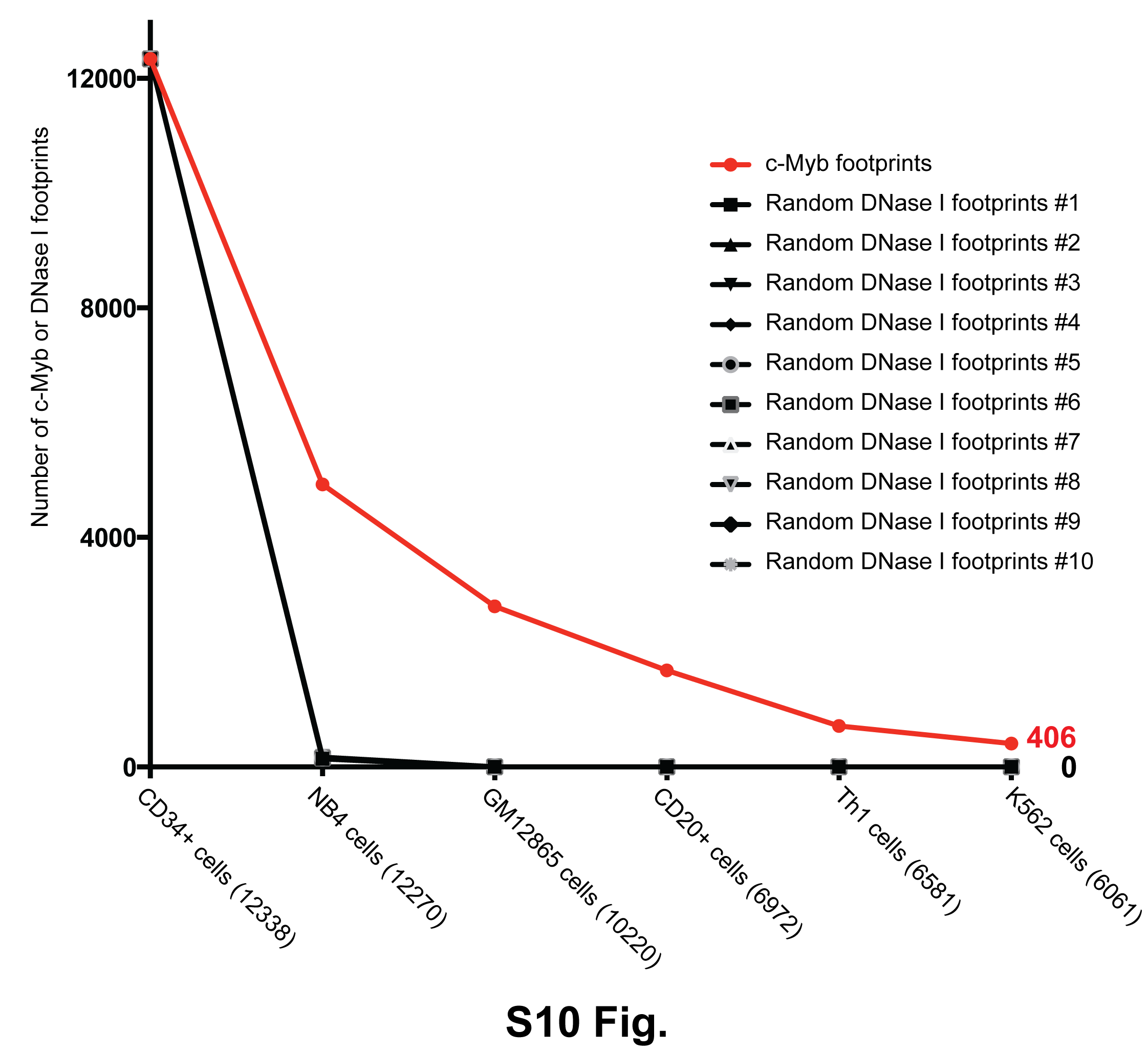

Supplement: S10 Fig — Graphs showing number of common c-Myb footprints or random selections of cell-specific DNase I footprints after subtraction of non-overlapping footprints between two cell-types at the time, and ending with the final number is a common set of footprints in all six cell-types. The analysis of a random selection of cell-specific DNase I footprints was repeated ten times starting with 12338 random footprints in CD34+ cells. The y-axis represents the number of c-Myb or DNase I footprints; the x-axis shows the six cell-types with total number of c-Myb footprints or number of random selection of cell-specific DNase I footprint used in the analysis (c-Myb footprints, red graph; random DNase I footprints, black bars). The numbers to the right indicate common footprints for c-Myb (red) or a random selection of cell-specific DNase I footprints (black) footprints common in all the cell-types. (TIF) [file pone.0133280.s010.tif]

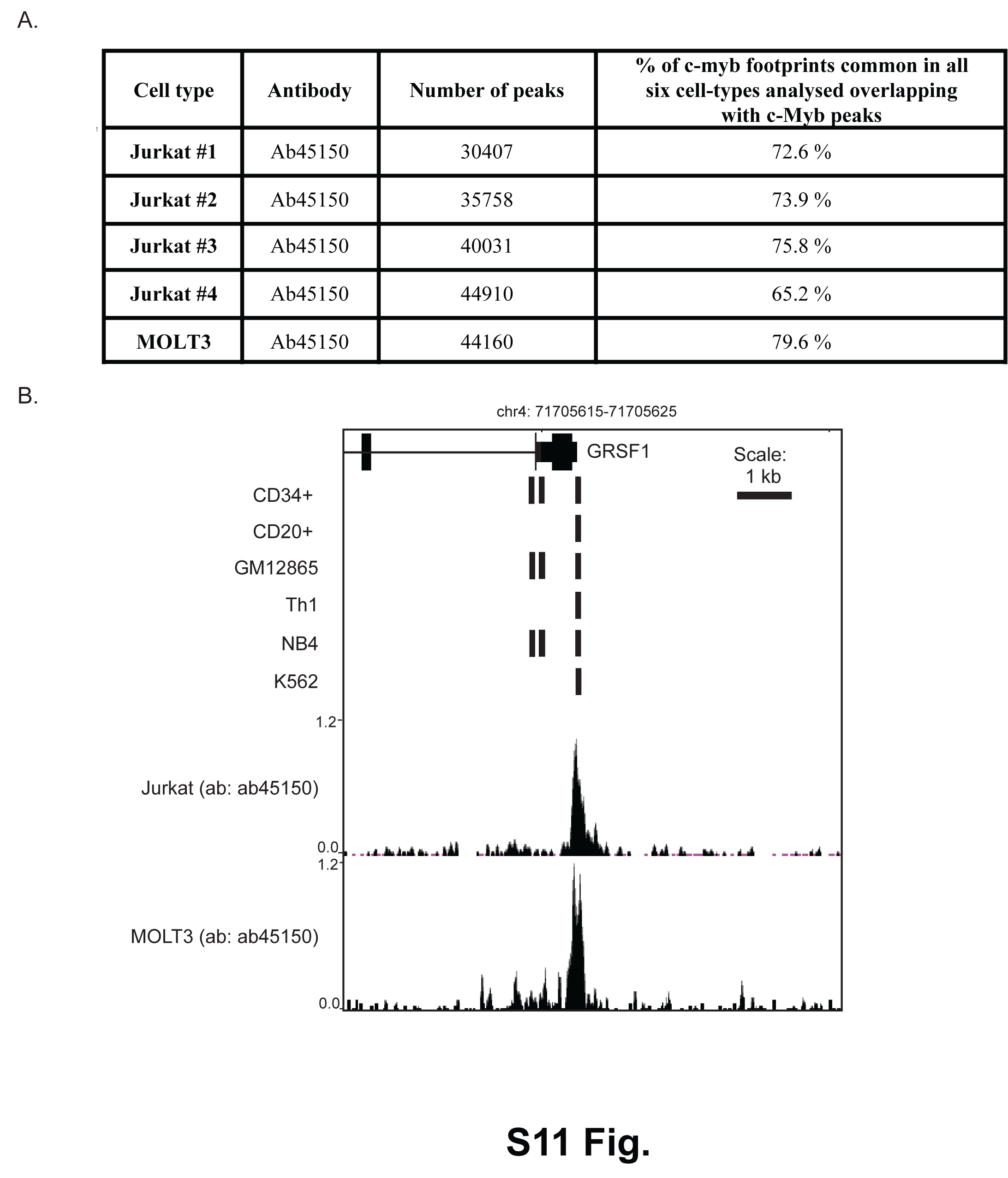

Supplement: S11 Fig — A) Overlap between c-Myb ChIP-Seq peaks for Jurkat and MOLT-3 cells [26] and the c-Myb footprints common in all the six cell-types analysed in this study. ChIP-Seq data was processed with SraTailor [94] using the default settings. B) An illustration showing the identified c-Myb common footprints at the promoter for GRSF1 for the six cell-types analysed in this study (see also Fig 1F) and enriched c-Myb ChIP-Seq signals for the same region in Jurkat and MOLT-3 cells. Coordinates for c-Myb footprint are shown above, and to the left are the signal intensities for the ChIP-Seq data shown. UCSC version hg19 (http://genome.ucsc.edu). (TIFF) [file pone.0133280.s011.tiff]
