# Supplementary material for: c-Myb Binding Sites in Haematopoietic Chromatin Landscapes
Source: PLoS One. 2015 Jul 24;10(7):e0133280. doi: 10.1371/journal.pone.0133280 (PMC4514710; doi:10.1371/journal.pone.0133280)
Supplement: S1 Table — The total number of footprints, footprints overlapping with c-Myb motifs and predicted c-Myb footprints in all the six cell-types analysed. (PDF) [file pone.0133280.s012.pdf]

S1 Table. DNaseI footprints and c-Myb footprints for the six cells types analysed. The total number of footprints, footprints overlapping with c-Myb motifs and predicted c-Myb footprints in all the six cell types analysed.

Cell type	Total number of digital footprints	Reference	Digital DNase I footprints with c-Myb motifs inside	Total number of c-Myb footprints
CD20+	603190	[1]	26809	6972
CD34+	902386	[1]	52767	12338
GM12865	811374	[1]	47676	10220
K562	498683	[1]	27060	6061
NB4	1049300	[1]	59086	12270
Th1	497983	[1]	27987	6581

Reference:

1. Neph S, Vierstra J, Stergachis AB, Reynolds AP, Haugen E, et al. (2012) An expansive human regulatory lexicon encoded in transcription factor footprints. Nature 489: 83–90. doi:10.1038/nature11212.
