# Supplementary material for: c-Myb Binding Sites in Haematopoietic Chromatin Landscapes
Source: PLoS One. 2015 Jul 24;10(7):e0133280. doi: 10.1371/journal.pone.0133280 (PMC4514710; doi:10.1371/journal.pone.0133280)
Supplement: S2 Table — Gene name, ID number, degree of regulation, if the gene contains a c-Myb footprint and the position of the footprint for the ten most downregulated genes in K562 cells upon knockdown of c-Myb [1]. (PDF) [file pone.0133280.s013.pdf]

S2 Table. The ten most downregulated genes in K562 cells upon knockdown of c-Myb. Gene name, ID number, degree of regulation, if the gene contains a c-Myb footprint and the position of the footprint for the ten most downregulated genes in K562 cells upon knockdown of c-Myb [1].

The promoter regions of the genes are defined as -2.5 kb upstream to +0.5 kb downstream of the TSS.

#	Gene Name	ENSG ID	Regulation upon KD of c-Myb in K562 cells (log2)	c-Myb footprint	Position of c-Myb footprints
1	KCNH2	ENSG00000055118	-1.128	Yes	Intragenic and promoter
2	LMO2	ENSG00000135363	-0.938	Yes	Intragenic and promoter
3	DSG1	ENSG00000134760	-0.904	No	-
4	CTSH	ENSG00000103811	-0.722	No	-
5	MYB	ENSG00000118513	-0.670	Yes	Intragenic and promoter
6	MYADM	ENSG00000179820	-0.631	Yes	Intragenic and promoter
7	GLUL	ENSG00000135821	-0.586	Yes	19 kb downstream of gene
8	STMN3	ENSG00000197457	-0.582	Yes	Promoter
9	EPCAM	ENSG00000119888	-0.569	Yes	Intragenic
10	GRSF1	ENSG00000132463	-0.547	Yes	Promoter

Reference:

1. Lorenzo PI, Brendeford EM, Gilfillan S, Gavrilov AA, Leedsak M, et al. (2011) Identification of c-Myb Target Genes in K562 Cells Reveals a Role for c-Myb as a Master Regulator. Genes & Cancer. doi:10.1177/1947601911428224.
