# Supplementary material for: c-Myb Binding Sites in Haematopoietic Chromatin Landscapes
Source: PLoS One. 2015 Jul 24;10(7):e0133280. doi: 10.1371/journal.pone.0133280 (PMC4514710; doi:10.1371/journal.pone.0133280)
Supplement: S3 Table — Gene name, ID number, degree of regulation, if the gene contains a c-Myb footprint and the position of the footprint for the ten most upregulated genes in K562 cells upon knockdown of c-Myb [1]. (PDF) [file pone.0133280.s014.pdf]

S3 Table. The ten most upregulated genes in K562 cells upon knockdown of c-Myb. Gene name, ID number, degree of regulation, if the gene contains a c-Myb footprint and the position of the footprint for the ten most upregulated genes in K562 cells upon knockdown of c-Myb [1]. The promoter regions of the genes are defined as -2.5 kb upstream to +0.5 kb downstream of the TSS.

#	Gene Name	ENSG ID	Regulation upon KD of c-Myb in K562 cells (log2)	c-Myb footprint	Position of c-Myb footprint
1	GYPB	ENSG00000250361	0.89	No	-
2	GDF15	ENSG00000130513	0.86	Yes	Promoter
3	MKRN1	ENSG00000133606	0.79	Yes	Promoter
4	SLC30A10	ENSG00000196660	0.78	Yes	15 kb upstream of TSS
5	MRAP2	ENSG00000135324	0.73	Yes	Intragenic
6	ALAS2	ENSG00000158578	0.73	No	-
7	BNIP3L	ENSG00000104765	0.72	No	-
8	LEPR	ENSG00000213625	0.65	Yes	Promoter
9	CPEB4	ENSG00000113742	0.65	Yes	Intragenic and promoter
10	SH3BGRL3	ENSG00000142669	0.62	Yes	3 kb upstream of TSS

Reference:

1. Lorenzo PI, Brendeford EM, Gilfillan S, Gavrilov AA, Leedsak M, et al. (2011) Identification of c-Myb Target Genes in K562 Cells Reveals a Role for c-Myb as a Master Regulator. Genes & Cancer. doi:10.1177/1947601911428224.
