# Supplementary material for: c-Myb Binding Sites in Haematopoietic Chromatin Landscapes
Source: PLoS One. 2015 Jul 24;10(7):e0133280. doi: 10.1371/journal.pone.0133280 (PMC4514710; doi:10.1371/journal.pone.0133280)
Supplement: S4 Table — FDR-corrected p-values, p', and normalised ratio for the distribution of c-Myb footprints at annotated genes, promoters and intergenic regions for the six cell-types analysed. (PDF) [file pone.0133280.s015.pdf]

S4 Table. Genomic localisation for c-Myb footprints in the six cell types analysed. FDR-**corrected p values, p', and normalised ratio** for the distribution of c-Myb footprints at annotated genes, promoters and intergenic regions for the six cell-types analysed.

**CD20+ cells**	**p'**	**Normalised ratio**
Intergenic regions	1.00	0.95
UTRs	0.52	1.10
Exons	1.87×10^{-3}	3.07
Introns	8.86×10^{-3}	1.08
Promoters	1.00	0.95
**CD34+ cells**		
Intergenic regions	1.00	0.91
UTRs	0.074	1.17
Exons	1.87×10^{-3}	3.52
Introns	1.00	0.98
Promoters	0.68	1,00
**GM12865 cells**		
Intergenic regions	1.00	0.87
UTRs	0.12	1.15
Exons	1.67×10^{-3}	3.96
Introns	1.00	0.95
Promoters	1.67×10^{-3}	1.04
**K562 cells**		
Intergenic regions	1.00	0.85
UTRs	0.043	1.25
Exons	9.37×10^{-4}	3.47
Introns	1.00	0.91
Promoters	9.37×10^{-4}	1.10
**NB4 cells**		
Intergenic regions	1.00	0.83
UTRs	0.12	1.13
Exons	9.37×10^{-4}	3.93
Introns	1.00	0.92
Promoters	9.37×10^{-4}	1.08
**Th1 cells**		
Intergenic regions	1.00	0.95
UTRs	2.25×10^{-3}	1.44
Exons	1.87×10^{-3}	2.98
Introns	0.069	1.06
Promoters	1.00	0.95
