# Supplementary material for: c-Myb Binding Sites in Haematopoietic Chromatin Landscapes
Source: PLoS One. 2015 Jul 24;10(7):e0133280. doi: 10.1371/journal.pone.0133280 (PMC4514710; doi:10.1371/journal.pone.0133280)
Supplement: S5 Table — Additional genes used in the study that are also regulated in K562 cells upon knockdown of c-Myb [1]. (PDF) [file pone.0133280.s016.pdf]

**S5 Table. Additional c-Myb target genes used in this study.** Additional genes used in the study that are also regulated in K562 cells upon knockdown of c-Myb [1].

The promoter regions of the genes are defined as -2.5 kb upstream to +0.5 kb downstream of the TSS.

| # | Gene Name | ENSG ID | Regulation upon KD of c-Myb in K562 cells (log2) | c-Myb footprint | Position of c-Myb footprints |
|---|---|---|---|---|---|
| 1 | BHLHE40 | ENSG00000134107 | 0.410 | Yes | Promoter |
| 2 | DCAF7 | ENSG00000136485 | -0.229 | Yes | Promoter |
| 3 | DUS3L | ENSG00000141994 | -0.275 | Yes | 11 kb upstream of TSS |
| 4 | RABEPK | ENSG00000136933 | -0.225 | Yes | Promoter |
| 5 | IKZF1 | ENSG00000185811 | -0.221 | Yes | Intragenic |
| 6 | SENP1 | ENSG00000079387 | -0.169 | Yes | Promoter |

**Reference:**

1. Lorenzo PI, Brendeford EM, Gilfillan S, Gavrilov AA, Leedsak M, et al. (2011) Identification of c-Myb Target Genes in K562 Cells Reveals a Role for c-Myb as a Master Regulator. Genes & Cancer. doi:10.1177/1947601911428224.
