# Supplementary material for: c-Myb Binding Sites in Haematopoietic Chromatin Landscapes
Source: PLoS One. 2015 Jul 24;10(7):e0133280. doi: 10.1371/journal.pone.0133280 (PMC4514710; doi:10.1371/journal.pone.0133280)
Supplement: S6 Table — FDR-corrected p-values, p', and normalised ratios for co-localisation between c-Myb footprints and ChIP-Seq peaks for the four histone modifications analysed in K562 cells. (PDF) [file pone.0133280.s017.pdf]

S6 Table. Co-localisation between c-Myb footprints and histone modifications. FDR-corrected p values , p', and normalised ratios for co-localization between c-Myb footprints and ChIP-Seq peaks for the four histone modifications analysed in K562 cells.

**Histone Modification**	**p'**	**Normalised ratio**
H3K4me3	4.00×10^{-4}	1.10
H3K4me1	4.00×10^{-4}	0.81
H3K9ac	0.31	1.01
H3K27me3	0.31	0.80
