# Supplementary material for: c-Myb Binding Sites in Haematopoietic Chromatin Landscapes
Source: PLoS One. 2015 Jul 24;10(7):e0133280. doi: 10.1371/journal.pone.0133280 (PMC4514710; doi:10.1371/journal.pone.0133280)
Supplement: S7 Table — Transcription factors with ChIP-seq peaks significantly overlapping c-Myb footprints at positively and negatively c-Myb-regulated genes. FDR-adjusted p-values, p', and normalised ratios are shown. For full list of ChIP-seq datasets analysed see S12 Table. (PDF) [file pone.0133280.s018.pdf]

S7 Table. Co-regulatory transcription factors. Transcription factors with ChIP-seq peaks significantly overlapping c-Myb footprints at positively and negatively c-Myb-regulated genes. FDR-adjusted p-values, p', and normalised ratios are shown.

For full list of ChIP-seq datasets analysed see S10 Table.

	**Genes with ChIP-Seq peak**			**Overlap with positively regulated genes**		**Overlap with negatively regulated genes**	
**Protein name**	**Positive**	**Negative**	**Total**	**p'**	**Normalised ratio**	**p'**	**Normalised ratio**
**Positive subset**							
SAP30	25	17	42	9.33×10^{-04}	3.10	0.05	1.63
SIN3A	23	16	39	9.33×10^{-04}	2.50	0.19	1.32
MXI1	24	17	41	9.33×10^{-04}	2.41	0.21	1.29
ETS1	26	20	46	9.33×10^{-04}	2.34	0.08	1.41
ELK1	11	11	22	1.57×10^{-02}	2.33	0.05	1.76
SIX5	12	9	21	2.37×10^{-02}	2.23	0.13	1.54
E2F4	20	18	38	$2{,}04 \times 10^{-03}$	2.00	0.21	1.29
PHF8	35	29	64	9.33×10^{-04}	1.93	0.04	1.38
RBBP5	25	21	46	9.48×10^{-03}	1.67	0.59	1.03
**Negative subset**							
THAP1	6	14	20	0.33	1.379	1.53×10^{-03}	2.81
