# Supplementary material for: c-Myb Binding Sites in Haematopoietic Chromatin Landscapes
Source: PLoS One. 2015 Jul 24;10(7):e0133280. doi: 10.1371/journal.pone.0133280 (PMC4514710; doi:10.1371/journal.pone.0133280)
Supplement: S8 Table — Gene list enriched in the four GO-terms (Fig 5B) identified by GREAT for c-Myb footprints common in all the six cell-types analysed in this study. The promoter regions of the genes are defined as -2.5 kb upstream to +0.5 kb downstream of the TSS. (PDF) [file pone.0133280.s019.pdf]

S8 Table: Genes enriched in the GO terms of c-Myb footprints common in all six cell-types. Gene list enriched in the four GO-terms (Fig. 5B) identified by GREAT for c-Myb footprints common in all the six cell-types analysed in this study. The promoter regions of the genes are defined as -2.5 kb upstream to +0.5 kb downstream of the TSS.

#	Gene name	Location of c-Myb footprint relative to gene	ENSG ID
1	ADAT2	Promoter	ENSG00000189007
2	APEX1	Promoter	ENSG00000100823
3	APLP1	+0.2 kb	ENSG00000105290
4	BRCA1	Promoter	ENSG00000012048
5	C22orf28	Promoter	ENSG00000100220
6	CASC3	Promoter	ENSG00000108349
7	CDKL3	+21 kb	ENSG00000006837
8	CLP1	Promoter	ENSG00000172409
9	CWC27	Promoter	ENSG00000153015
10	DCAF13	Promoter	ENSG00000164934
11	DCP1B	Promoter	ENSG00000151065
12	DDX23	Promoter	ENSG00000174243
13	DDX41	Promoter	ENSG00000183258
14	ERCC1	Intragenic	ENSG00000012061
15	EXOSC5	Promoter	ENSG00000077348
16	FBL	-35 kb	ENSG00000105202
17	FDXACB1	Promoter	ENSG00000255561
18	GRSF1	Promoter	ENSG00000132463
19	HNRNPA0	Promoter	ENSG00000177733
20	HNRNPA1	Promoter	ENSG00000135486
21	HNRNPA3	+40 kb	ENSG00000170144
22	HNRNPUL1	Promoter	ENSG00000105323
23	HSD17B10	-250 kb	ENSG00000072506
24	INTS7	Promoter	ENSG00000143493
25	KIN	Promoter	ENSG00000151657
26	KLHDC3	Promoter	ENSG00000124702
27	LSM4	-5 kb	ENSG00000130520
28	MBNL1	Promoter	ENSG00000152601
29	MDC1	Promoter	ENSG00000137337
30	MUS81	Promoter	ENSG00000172732
31	NONO	Promoter	ENSG00000147140
32	OSGEP	Promoter	ENSG00000092094
33	PCBP1	-16 kb	ENSG00000169564
34	POLD4	Promoter	ENSG00000175482
35	POLR2A	Promoter	ENSG00000181222
36	POLR2L	Promoter	ENSG00000177700

S8 Table continued.

37	POP5	Promoter	ENSG00000167272
38	PPAN-P2RY11	Promoter	ENSG00000243207
39	PPIA	Promoter	ENSG00000196262
40	PSMB4	+42 kb	ENSG00000159377
41	PSME2	Promoter	ENSG00000100911
42	PTBP1	Promoter	ENSG00000011304
43	RAD21	Promoter	ENSG00000164754
44	RBM14	Promoter	ENSG00000239306
45	RBM6	Promoter	ENSG00000004534
46	RBPJ	Promoter	ENSG00000168214
47	RFC5	Promoter	ENSG00000111445
48	RNMT	Promoter	ENSG00000101654
49	RPL26	Promoter	ENSG00000161970
50	RPL37A	Promoter	ENSG00000197756
51	RPL9	-65 kb	ENSG00000237550
52	RPS28	+20 kb	ENSG00000233927
53	RPS29	Promoter	ENSG00000213741
54	RPS8	Promoter	ENSG00000142937
55	RUVBL2	Promoter	ENSG00000183207
56	SMNDC1	+80 kb	ENSG00000119953
57	SNRNP48	Promoter	ENSG00000168566
58	SREK1IP1	Promoter	ENSG00000153006
59	SRSF11	Promoter	ENSG00000116754
60	SRSF3	+18 kb	ENSG00000112081
61	TCF7	Intragenic	ENSG00000081059
62	UBE2N	Promoter	ENSG00000177889
63	UPF2	Intragenic	ENSG00000151461
64	UTP14A	Promoter	ENSG00000156697
65	VEGFA	+293 kb	ENSG00000112715
