# Supplementary material for: c-Myb Binding Sites in Haematopoietic Chromatin Landscapes
Source: PLoS One. 2015 Jul 24;10(7):e0133280. doi: 10.1371/journal.pone.0133280 (PMC4514710; doi:10.1371/journal.pone.0133280)
Supplement: S9 Table — (PDF) [file pone.0133280.s020.pdf]

S9 Table. Overlap between suggested co-regulatory factors in K562 cells and c-Myb footprints common in all six-cell types.

**Factor**	**c-Myb footprints common in all six cell types overlapping with ChIP-seq peaks for the factor**
**Positive set**	
ETS1	123
SAP30	101
E2F4	134
ELK1	64
SIX5	58
PHF8	204
SIN3A	133
RBBP5	139
MXI1	116
**Negative set**	
THAP1	55
