# Supplementary material for: c-Myb Binding Sites in Haematopoietic Chromatin Landscapes
Source: PLoS One. 2015 Jul 24;10(7):e0133280. doi: 10.1371/journal.pone.0133280 (PMC4514710; doi:10.1371/journal.pone.0133280)
Supplement: S10 Table — (PDF) [file pone.0133280.s021.pdf]

S10 Table. List of primers used in this study.

**Primers for cloning**		**Ref.**
DCAF7_L_F	cactgatgctagcaCAGACGCTACGGCCGCAATA	
DCAF7_L_R	tagcatgactcgagTGAGATCTATGGGTCCGAAG	
DUS3L_L_F	ctctgatgctagcaCACTCCTCGTTCCAAAGCT	
DUS3L_L_R	cagtacttctcgagTGCAGTCCAGTTCGCAATTG	
IKZF1_L_F	ctctgatgctagcaCACTCTGCTAAGCTACTCAA	
IKZF1_L_R	acactagactcgagTCCCTCCATATCCAGAGACA	
KB-1458E12.1_L_F	ctctgatgctagcaCTGGTATCCCCACATTCTAA	
KB-1458E12.1_L_R	cagtacttctcgagTGGACTTTGAAACGCTGCCATG	
KCHN2#1_L_F	ctctgatgctagcaCAAAGGTAGGGCTAGAATCCA	
KCHN2#1_L_R	cagtacttctcgagTCATGTGCCTGTCCCTAAGG	
KCNH2#2_L_F	ctctgatgctagcaGTCCCTTCACTTGCCCCAGT	
KCNH2#2_L_R	acgtactactcgagTGAGACTGGGAACCCTCTCC	
LMO2_L_F	ctctgatgctagcaCTACTTTGACCTGAGTGTGTA	
LMO2_L_R	acactagactcgagTACAATAAGGTCATGATTGC	
MYADM_L_F	ctctgatgctagcaCTCTGCACTAAACAAACCCTA	
MYADM_L_R	acgtacacctcgagAGAGTAAAAGTAGCGTGGAG	
C10orf55_L_F	acttgatgctagcaCTGACATGCCCGCAGAATCCA	
C10orf55_L_R	agatatacactcgagTGAAGAGCTGGAGCCTCAGG	
RABEPK_L_F	ctctgatgctagcaAGCCTGTTCTTTCCCGACC	
RABEPK_L_R	cagacgatctcgagTGGACCTGGCATGGAGTAG	
RUNX2_L_F	ctctgatgctagcaCACCTCTTCCTGTCTTGTAACA	
RUNX2_L_R	cagtacttctcagTGAGCTAAAAGCGACGAATAAG	
SENP1_L_F	acttgatgctagcaGACACAGTCTCCTGGACCA	
SENP1_L_F	acactagactcgagCAGACTGAAAAGGGTACTGAG	
Control#1_L_F	ctctgatgctagcaCTGGTATGATAGAACTGTCC	
Control#1_L_R	cagtacttctcagTATGAAAGAGCAAAGCAAGG	
GRSF1_L_F	ctctgatgctagcaATAGAGCCAGCGGCGGAGTA	
GRSF1_L_R	TCCTCCACCTCCTTCCGACTC	
RUNX1#1_L_F	ctctgatgctagcaTAACTGTCACCAAGGGCTGGGA	
RUNX1#1_L_R	cagacgatctcgagATGCAAAAGAAGTTCAAAGGCTGA	
**Primers for DamID**		
IKZF1_F	CTGGTATCTCGCGGCGGCTTCC	
IKZF1_R	ACACTGTGCTTGTGTGTCATC	
PA2G4_F	CTCAGCGTTCTCGGTGGAAGT	
PA2G4_R	CGGAATCGCCGGGCACTCTG	[1]
RUNX1_F	GATACAGCAAGGTCGTTGCTCA	
RUNX1_R	ACCCAGGAAACACCTTGCAGAG	
MYADM_F	GCTGTTGTGTCTTCCTGTTTTGT	
MYADM_R	CGCCCCCCAGAGTAAAAGT	
LMO2_F	CTGCATATGACTTTGGGTCA	
LMO2_R	GAATGAAATCCCACCGGTAG	

KCHN2_F	CACAATACTGACCATCGGAC	
KCHN2_R	AGTTGGGAGAGGATGACATT	
CBFA2T3_F	CACTCCCCACCTCCCCACTA	
CBFA2T3_R	CTGAAAGCCACAGCCAACGTC	
BHLHE40_F	CCCTTTCCAACCCAGTCCTT	
BHLHE40_T	GGTTCTCGCCTCATTTGCAT	
Control#1_F	CCTGGTATGCACCAACACAG	[2]
Control#1_R	TTCAGGAGTCTGGGGATTTG	[2]
Control#2_F	CATGACACTAGTTGTGCTGTG	
Control#2_R	CTGTCCTATGTTGGCTCAGAT	
GRSF1_F	ATAGAGCCAGCGGCGGAGTA	
GRSF1_R	CAACCGGCCCTGGATTCCAC	

Reference:

1. Zhao L, Glazov EA, Pattabiraman DR, Al-Owaidi F, Zhang P, et al. (2011) Integrated genome-wide chromatin occupancy and expression analyses identify key myeloid pro-differentiation transcription factors repressed by Myb. Nucleic Acids Res 39: 4664–4679. doi:10.1093/nar/gkr024.

2. Barski A, Cuddapah S, Cui K, Roh T-Y, Schones DE, et al. (2007) High-resolution profiling of histone methylations in the human genome. Cell 129: 823–837. doi:10.1016/j.cell.2007.05.009.
