# Supplementary material for: c-Myb Binding Sites in Haematopoietic Chromatin Landscapes
Source: PLoS One. 2015 Jul 24;10(7):e0133280. doi: 10.1371/journal.pone.0133280 (PMC4514710; doi:10.1371/journal.pone.0133280)
Supplement: S11 Table — (PDF) [file pone.0133280.s022.pdf]

S11 Table. Antibodies used in this study.

**Antibody**	**Dilution for Western analysis**
Anti-FLAG M2 antibody (mouse, monoclonal) (F3165) (Sigma-Aldrich)	1:5000
Anti-c-Myb antibody (rabbit, polyclonal) (H-141) (Santa Cruz Biotech)	1:200
Anti-Gapdh antibody (mouse, monoclonal) (AM4300) (Invitrogen)	1:10000
