# Supplementary material for: c-Myb Binding Sites in Haematopoietic Chromatin Landscapes
Source: PLoS One. 2015 Jul 24;10(7):e0133280. doi: 10.1371/journal.pone.0133280 (PMC4514710; doi:10.1371/journal.pone.0133280)
Supplement: S12 Table — (PDF) [file pone.0133280.s023.pdf]

S12 Table. List of ChIP-Seq peak data sets used in this study.

#	Factor	Number of peaks	Source lab	Reference
1	ARID3A	9026	Snyder	ENCODE
2	ATF1	14864	Struhl/Snyder	ENCODE
3	ATF3	1233	Struhl/Snyder	ENCODE
4	BACH1	3806	Snyder	ENCODE
5	BCL3	1603	Myers	ENCODE
6	BCLAF1	4444	Myers	ENCODE
7	BDP1	570	Struhl/Snyder	ENCODE
8	BHLHE40	22497	Snyder	ENCODE
9	BRF1	221	Struhl/Snyder	ENCODE
10	BRF2	1087	Struhl/Snyder	ENCODE
11	BRG1	2463	Snyder	ENCODE
12	c-FOS	7646	Snyder	ENCODE
13	c-JUN	9848	Snyder	ENCODE
14	CBX3	20723	Myers	ENCODE
15	CCNT2	20057	Struhl/Snyder	ENCODE
16	CEBP	38715	Snyder	ENCODE
17	CHD1	9350	Bernstein	ENCODE
18	CHD2	7797	Snyder	ENCODE
19	CHD4	13735	Bernstein	ENCODE
20	CHD7	30660	Bernstein	ENCODE
21	COREST	35741	Snyder	ENCODE
22	CTCFL	54387	Myers	ENCODE
23	CTCFL	11533	Myers	ENCODE
24	E2F4	8181	Farnham/Snyder	ENCODE
25	E2F6	16312	Farnham/Snyder	ENCODE
26	eGFP-FOS	10256	White	ENCODE
27	eGFP-JUNB	12287	White	ENCODE
28	EGR1	36997	Myers	ENCODE
29	ELF1	27780	Myers	ENCODE
30	ELK1	2961	Snyder	ENCODE
31	ETS1	10726	Myers	ENCODE
32	EZH2	1685	Bernstein	ENCODE
33	FOSL1	11174	Myers	ENCODE
34	GABP	14393	Myers	ENCODE
35	GATA1	4074	Farnham/Snyder	ENCODE
36	GATA2	10648	Farnham/Snyder	ENCODE
37	GTF2B	2928	Struhl/Snyder	ENCODE
38	GTF2F1	3621	Snyder	ENCODE
39	HCFC1	42132	Snyder	ENCODE
40	HDAC1	11833	Bernstein	ENCODE
41	HDAC2	5247	Bernstein	ENCODE
42	HDAC6	1114	Bernstein	ENCODE

43	HDAC8	1718	White	ENCODE
44	HMGN3	14587	Struhl/Snyder	ENCODE
45	INI1	1942	Snyder	ENCODE
46	JUND	40052	Snyder	ENCODE
47	KAP1	5489	Farnham/Snyder	ENCODE
48	KDM5B	15520	Bernstein	ENCODE
49	MAFF	25074	Snyder	ENCODE
50	MAFK	19317	Snyder	ENCODE
51	MAX	31436	Snyder	ENCODE
52	MAZ	33323	Snyder	ENCODE
53	MEF2	5631	Myers	ENCODE
54	MXI1	6711	Snyder	ENCODE
55	MYC	5023	Snyder	ENCODE
56	MYC	24153	Snyder	ENCODE
57	NELFE	458	Struhl/Snyder	ENCODE
58	NFE2	2637	Myers	ENCODE
59	NFYA	4286	Snyder	ENCODE
60	NFYB	10096	Snyder	ENCODE
61	NR2C2	587	Farnham/Snyder	ENCODE
62	NR2F2	16678	Myers	ENCODE
63	NR4A1	7883	White	ENCODE
64	NRF1	4211	Snyder	ENCODE
65	NRSF	15849	Myers	ENCODE
66	P300	76018	Snyder	ENCODE
67	P300	25881	Snyder	ENCODE
68	PCAF	16253	Bernstein	ENCODE
69	PHF8	22302	Bernstein	ENCODE
70	PML	15895	Myers	ENCODE
71	POL2	18081	Snyder	ENCODE
72	POL2S2	5397	Snyder	ENCODE
73	POL3	207	Snyder	ENCODE
74	RAD21	17627	Snyder	ENCODE
75	RBBP5	14258	Bernstein	ENCODE
76	RFX5	2201	Snyder	ENCODE
77	RPC155	131	Struhl/Snyder	ENCODE
78	SAP30	9753	Bernstein	ENCODE
79	SETDB1	3882	Farnham/Snyder	ENCODE
80	SIN3A	12700	Myers	ENCODE
81	SIRT6	2305	Struhl/Snyder	ENCODE
82	SIX5	4194	Myers	ENCODE
83	SMC3	23598	Snyder	ENCODE
84	SP1	7206	Myers	ENCODE
85	SP2	3124	Myers	ENCODE
86	SPI1	28677	Myers	ENCODE
87	SRF	4717	Myers	ENCODE

88	STAT5A	9811	Myers	ENCODE
89	TAF1	15246	Myers	ENCODE
90	TAF7	3422	Myers	ENCODE
91	TAL1	26260	Snyder	ENCODE
92	TBL1XR1	5086	Snyder	ENCODE
93	TBP	17558	Snyder	ENCODE
94	TEAD4	31030	Myers	ENCODE
95	TFIIIC	1858	Struhl/Snyder	ENCODE
96	THAP1	3506	Myers	ENCODE
97	TRIM28	12137	Myers	ENCODE
98	UBTF	6002	Snyder	ENCODE
99	UBTF	13692	Snyder	ENCODE
100	USF1	18521	Myers	ENCODE
101	USF2	3083	Snyder	ENCODE
102	YY1	4948	Farnham/Snyder	ENCODE
103	ZBTB33	3285	Myers	ENCODE
104	ZBTB7A	21711	Myers	ENCODE
105	ZNF143	29069	Snyder	ENCODE
106	ZNF263	3081	Farnham/Snyder	ENCODE
107	ZNF274	1997	Farnham/Snyder	ENCODE
**#**	**Histone modification**	**Number of peaks**	**Souce lab**	**Reference**
1	H3K4me3	18662	Farnham/Snyder	ENCODE
2	H3K4me1	108851	Farnham/Snyder	ENCODE
3	H3K9ac	36409	Farnham/Snyder	ENCODE
4	H3K27me3	134768	Farnham/Snyder	ENCODE
